# Panorama Phylogenetic Diversity and Distribution of Type A Influenza Virus

**DOI:** 10.1371/journal.pone.0005022

**Published:** 2009-03-27

**Authors:** Shuo Liu, Kang Ji, Jiming Chen, Di Tai, Wenming Jiang, Guangyu Hou, Jie Chen, Jinping Li, Baoxu Huang

**Affiliations:** 1 The Laboratory of Animal Epidemiological Surveillance, China Animal Health & Epidemiology Center, Qingdao, China; 2 College of Veterinary Sciences, University of Northeastern China, Harbin, China; NERC Centre for Ecology and Hydrology, United Kingdom

## Abstract

**Background:**

Type A influenza virus is one of important pathogens of various animals, including humans, pigs, horses, marine mammals and birds. Currently, the viral type has been classified into 16 hemagglutinin and 9 neuraminidase subtypes, but the phylogenetic diversity and distribution within the viral type largely remain unclear from the whole view.

**Methodology/Principal Findings:**

The panorama phylogenetic trees of influenza A viruses were calculated with representative sequences selected from approximately 23000 candidates available in GenBank using web servers in NCBI and the software MEGA 4.0. Lineages and sublineages were classified according to genetic distances, topology of the phylogenetic trees and distributions of the viruses in hosts, regions and time.

**Conclusions/Significance:**

Here, two panorama phylogenetic trees of type A influenza virus covering all the 16 hemagglutinin subtypes and 9 neuraminidase subtypes, respectively, were generated. The trees provided us whole views and some novel information to recognize influenza A viruses including that some subtypes of avian influenza viruses are more complicated than Eurasian and North American lineages as we thought in the past. They also provide us a framework to generalize the history and explore the future of the viral circulation and evolution in different kinds of hosts. In addition, a simple and comprehensive nomenclature system for the dozens of lineages and sublineages identified within the viral type was proposed, which if universally accepted, will facilitate communications on the viral evolution, ecology and epidemiology.

## Introduction

Influenza A virus causes frequent epidemics and occasional pandemics in various animals, including humans, pigs, horses, marine mammals and birds [1]. The viral genome comprises 8 segments which totally encode at least 10 proteins. The fourth and sixth segments encode the viral hemagglutinin (HA) and neuraminidase (NA), respectively, both of which are highly variable and diversified. Currently, 16 HA subtypes (H1∼H16) and 9 NA subtypes (N1∼N9) of influenza A virus have been identified [2]. Their combination further generates dozens of subtypes of influenza A virus, like H1N1, H3N2, H7N7, H9N2, etc.

Till date, the phylogenetic diversity and distribution of influenza A viruses have been widely studied within some regions, periods, hosts or viral lineages, but seldom detailed within the type or a subtype from the panorama view except for subtypes H5 and N1 influenza virus [1]–[39]. Moreover, most of the 16 HA and 9 NA subtypes have evolved into some distinct lineages and sublineages, but it is unknown that how many lineages and sublineages exist within most of the subtypes. In addition, some of the lineages and sublineages have been designated as the North American lineage, swine human-like H1 viruses, A/chicken/Hong Kong/G9/97(H9N2)-like viruses, Fujian-like viruses, etc., as required by the studies on evolution, ecology and epidemiology of influenza A virus. These nomenclatures are ambiguous, miscellaneous, self-limited and sometimes even misleading. Therefore, it is of significance to identify all the lineages and sublineages within type A influenza virus and unify their nomenclature with a more rational system.

In this report, approximately 15000 HA gene sequences and 8000 NA gene of influenza A viruses available in GenBank were analyzed along with their background in order to identify all the lineages and sublineages as well as elucidate the panorama phylogenetic diversity and distribution within the whole viral type covering all the 16 HA and 9 NA subtypes. Meanwhile, a simple and comprehensive nomenclature system for the lineages and sublineages was also proposed, which if universally accepted, will facilitate international communications on the viral evolution, ecology and epidemiology.

## Results

### Host and subtype distributions of type A influenza virus

Up to July 11, 2008, HA sequences of 14328 (2377 H1, 163 H2, 7425 H3, 132 H4, 2397 H5, 374 H6, 466 H7, 12 H8, 800 H9, 59 H10, 65 H11, 24 H12, 15 H13, 3 H14, 6 H15 and 10 H16) influenza viruses with clear background, and NA sequences of 7836 (3100 N1, 3967 N2, 192 N3, 26 N4, 58 N5, 125 N6, 75 N7, 224 N8, 69 N9) influenza viruses with clear background were available in GenBank, after excluding the sequences of unclear background, the same viruses or manipulated materials and the ones shorter than 500 bp or with sequencing errors. Their subtype distribution was further detailed in Table 1 and Table 2.

**Table 1:** NA-subtype distribution of 14328 influenza A viruses with HA sequences in GenBank*.

Subtypes	N1	N2	N3	N4	N5	N6	N7	N8	N9	Unknown	Total
H1	***2162***	***148***	1	0	2	3	1	0	2	58	2377
H2	11	***99***	23	1	8	0	3	4	12	2	163
H3	17	***6658***	11	1	4	29	0	***231***	2	472	7425
H4	4	16	5	3	3	***69***	2	23	2	5	132
H5	***2103***	***204***	49	0	1	3	9	4	14	10	2397
H6	***148***	***123***	6	6	21	8	1	45	5	11	374
H7	***107***	***156***	***94***	4	1	0	***83***	3	3	15	466
H8	0	1	0	***11***	0	0	0	0	0	0	12
H9	10	***773***	3	0	4	3	0	1	0	6	800
H10	2	1	6	3	0	3	***38***	3	2	1	59
H11	3	10	6	1	0	2	0	2	***38***	3	65
H12	1	2	0	2	16	0	0	0	2	1	24
H13	0	4	0	0	0	9	0	1	1	0	15
H14	0	0	0	0	1	0	0	0	0	2	3
H15	0	1	0	0	0	0	0	1	4	0	6
H16	0	0	10	0	0	0	0	0	0	0	10

* The presumably predominant NA subtypes within each HA subtype were in bold and italics.

**Table 2:** HA-subtype distribution of 7892 influenza A viruses with NA sequences in GenBank*.

Subtypes	N1	N2	N3	N4	N5	N6	N7	N8	N9
H1	***1329***	***109***	2	0	2	3	1	0	2
H2	9	***87***	17	1	8	0	3	0	9
H3	29	***2662***	10	0	5	30	2	***154***	2
H4	4	14	5	3	3	***67***	3	17	2
H5	***1463***	***124***	43	0	0	1	0	2	2
H6	***155***	***96***	6	1	12	8	0	34	3
H7	***55***	***165***	***78***	3	1	3	***29***	3	1
H8	0	0	1	***11***	0	0	0	0	0
H9	9	***693***	2	0	3	0	0	1	0
H10	2	0	16	3	4	3	***37***	6	2
H11	7	14	6	2	0	3	0	2	***37***
H12	1	0	0	2	19	0	0	0	2
H13	0	3	1	0	0	7	0	1	1
H14	0	0	0	0	1	0	0	0	0
H15	0	0	0	0	0	0	0	1	0
H16	0	0	6	0	0	0	0	0	0
Unknown	25	75	0	0	0	0	0	0	1
Total	3088	4042	193	26	58	125	75	221	64

* The presumably predominant HA subtypes within each NA subtype were consistent with Table 1 and in bold and italics.

Among the 14328 influenza A viruses with HA sequences in GenBank, 4383 (2120 H5, 759 H9, 446 H7, 374 H6, 215 H3, 129 H4, 77 H2, 71 H1, 65 H11, 58 H10, 24 H12, 14 H13, 12 H8, 10 H16, 6 H15 and 3 H14) were from birds, 9199 (6868 H3, 1994 H1, 240 H5, 84 H2, 7 H9 and 6 H7) were from humans; 539 (311 H1, 179 H3, 34 H9, 11 H5, 2 H2, 2 H4) were from pigs; 166 (154 H3, 12 H7) were from horses; 41 were from other hosts including 7 H3 from dogs, 2 H3 and 1 H4 from seals;11 H5 from tigers, 9 H5 from cats, 3 H5 from leopards, 1 H5 from a stone marten, 1 H1 from a giant anteater, 1 H5 from a civet, 1 H5 from a dog, 1 H5 from a blow fly, 2 H7 from seals and 1 H13 from a whale.

Among the 7836 influenza A viruses with NA sequences in GenBank, 3446 (1602 N1, 1167 N2, 187 N3, 147 N8, 124 N6, 68 N7, 68 N9, 58 N5, 25 N4) were from birds, 3905 (2617 N2, 1285 N1, 2 N7, 1 N3) were from humans; 377 (188 N1, 183 N2, 4 N3, 1 N6, 1 N7) were from pigs; 77 (74 N8, 3 N7) were from horses; 31 were from other hosts including 11 N1 from tigers, 5 N1 from cats, 1 N1 and 3 N8 from dogs; 3 N1 from leopards, 1 N1 from a blow fly, 1 N1 from a civet, 1 N1 from a stone marten, 1 N1 from a giant anteater, 1 N1 from a mouse, 1 N4 from a mink, 1 N7 from a seal and 1 N9 from a whale.

The host distribution of the viruses was also given in Table 3 and Table 4.

**Table 3:** Host distribution of 14328 influenza A viruses with HA sequences in GenBank*

	Birds	Humans	Pigs	Horses	Others
H1	71	***1994***	***311***	0	1
H2	77	84	2	0	0
H3	***215***	***6868***	***179***	***154***	9
H4	129	0	2	0	1
H5	***2120***	***240***	11	0	***27***
H6	***374***	0	0	0	0
H7	***446***	6	0	12	2
H8	12	0	0	0	0
H9	***759***	7	34	0	0
H10	58	0	0	0	0
H11	65	0	0	0	0
H12	24	0	0	0	0
H13	14	0	0	0	1
H14	3	0	0	0	0
H15	6	0	0	0	0
H16	10	0	0	0	0

* The presumably predominant HA subtypes circulating in the hosts were in bold and italics.

**Table 4:** Host distribution of 7836 influenza A viruses with NA sequences in GenBank*

	Birds	Humans	Pigs	Horses	Others
N1	***1602***	***1285***	***188***	0	***25***
N2	***1167***	***2617***	***183***	0	0
N3	187	1	4	0	0
N4	25	0	0	0	1
N5	58	0	0	0	0
N6	124	0	1	0	0
N7	68	2	1	3	1
N8	147	0	0	***74***	3
N9	68	0	0	0	1

* The presumably predominant NA subtypes circulating in the hosts were marked with in bold and italics.

### Panorama phylogenetic diversity and distribution of type A influenza virus based on HA sequences

**Figure 1:**
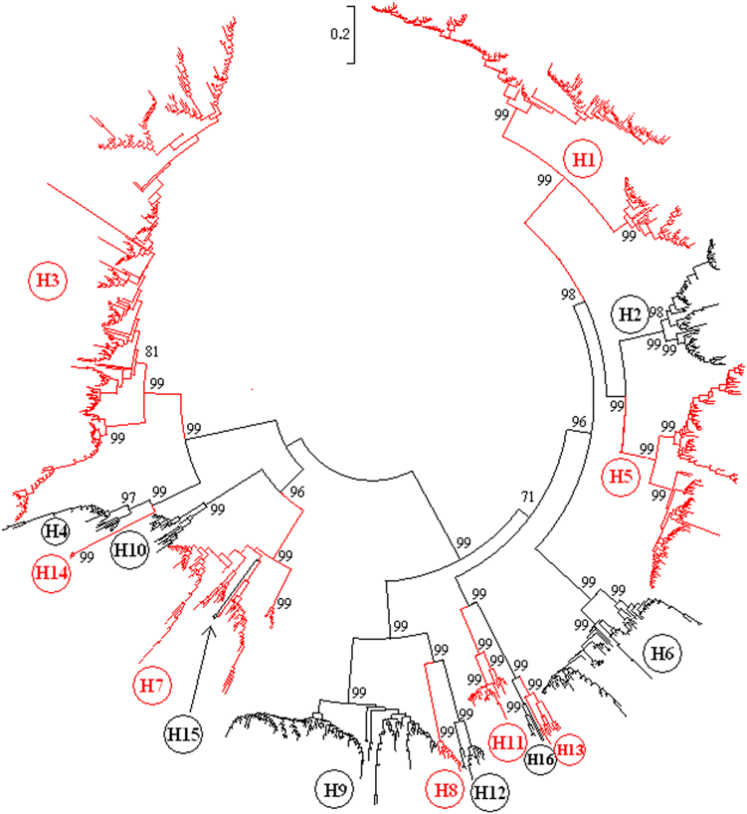
The panorama phylogenetic tree of type A influenza virus based on the viral HA sequences. The tree demonstrated that subtype H16 is close to subtype H13, and subtype H15 is similar to a lineage within subtype H7. The panorama diversity of each of the HA subtypes was detailed in Figure 3∼[Fig pone-0005022-g004] [Fig pone-0005022-g005] [Fig pone-0005022-g006] [Fig pone-0005022-g007] [Fig pone-0005022-g008] Figure 9. Bootstrap values were given at relevant nodes.

1264 representatives were selected from the 14328 influenza A viruses with HA sequences in GenBank. The designations of the representative viruses were partially demonstrated in Table S1 and are available upon request. The phylogenetic tree of the representatives based on their HA sequences provided us a panorama view to the phylogenetic diversity and distribution of type A influenza virus (Figure 1). First, most of the 16 HA subtypes could be divided into some distinct lineages which were detailed in the following sections and generalized in Table S1. Second, most of the 16 HA subtypes were distant to each other in genetics except the two pairs, H13 and H16, H7 and H15. H15 was so close to H7 that it looked like a lineage of subtype H7.

Among the 16 HA subtypes, the average genetic distance between H3 and H13 was largest ( = 213.9%, ranging from 160.6% to 383.3%), and that between H13 and H16 was smallest ( = 44.3%, ranging from 40.6% to 50.6%).

Among the 16 HA subtypes, only some strains of subtypes H5 and H7 influenza viruses were found with 3 or more basic amino acid residues at the cleavage site of the HA protein which have been known a premise for high pathogenicity in birds [3]. This is consistent with the fact that only some H5 and H7 influenza viruses were highly pathogenic to birds. Among the thousands of mammalian influenza A viruses, only and all known equine H7N7 influenza viruses harbored 3 or more basic amino acid residues at the cleavage site of the HA protein.

### Panorama phylogenetic diversity and distribution of type A influenza virus based on NA sequences

**Figure 2:**
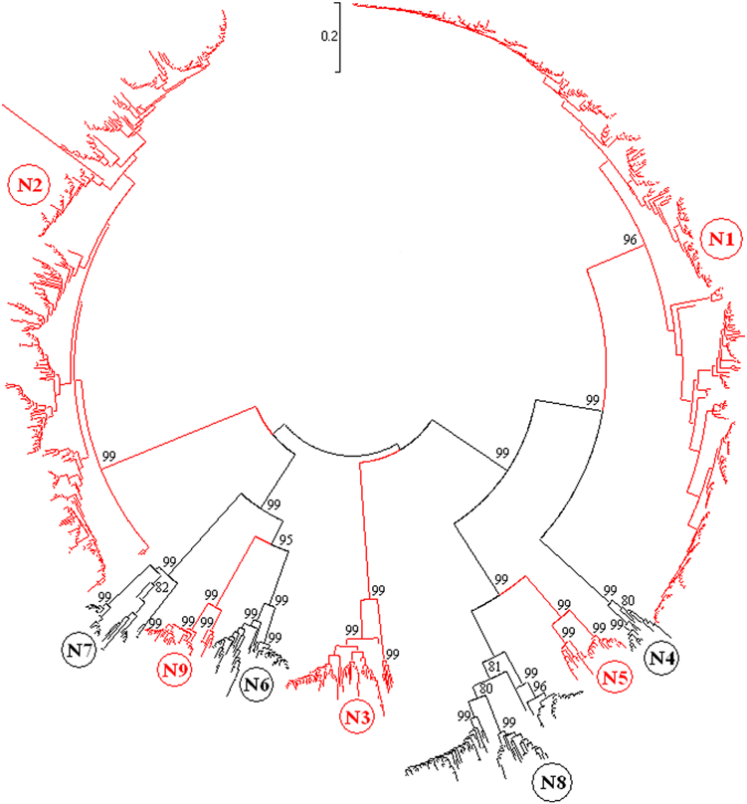
The panorama phylogenetic tree of type A influenza virus based on the viral NA representative sequences. The tree demonstrated that all the 9 NA subtypes were genetically distant to each other, and subtypes N1 and N2 were more dominant than others. The panorama diversity of each of the NA subtypes was detailed in Figure 10∼[Fig pone-0005022-g011] [Fig pone-0005022-g012] [Fig pone-0005022-g013] Figure 14. Bootstrap values were given at relevant nodes.

1154 representatives were selected from the 7836 influenza A viruses with HA sequences in GenBank. The designations of the representative viruses were partially demonstrated in Table S2 are available upon request. The phylogenetic tree of the representatives based on their NA sequences provided us another panorama view to the phylogenetic diversity and distribution of type A influenza virus (Figure 2). First, most of the 9 subtypes could be divided into some distinct lineages which were detailed in the following sections and generalized in Table S2. Second, all the 9 subtypes are distant to each other in genetics.

Among the 9 NA subtypes, the average genetic distance between N4 and N9 was largest ( = 176.4% ranging from 155.1% to 200.4%), and that between N5 and N8 was smallest ( = 66.7% ranging from 57.3% to 81.5%).

### The phylogenetic distribution of H1 subtype influenza viruses

**Figure 3:**
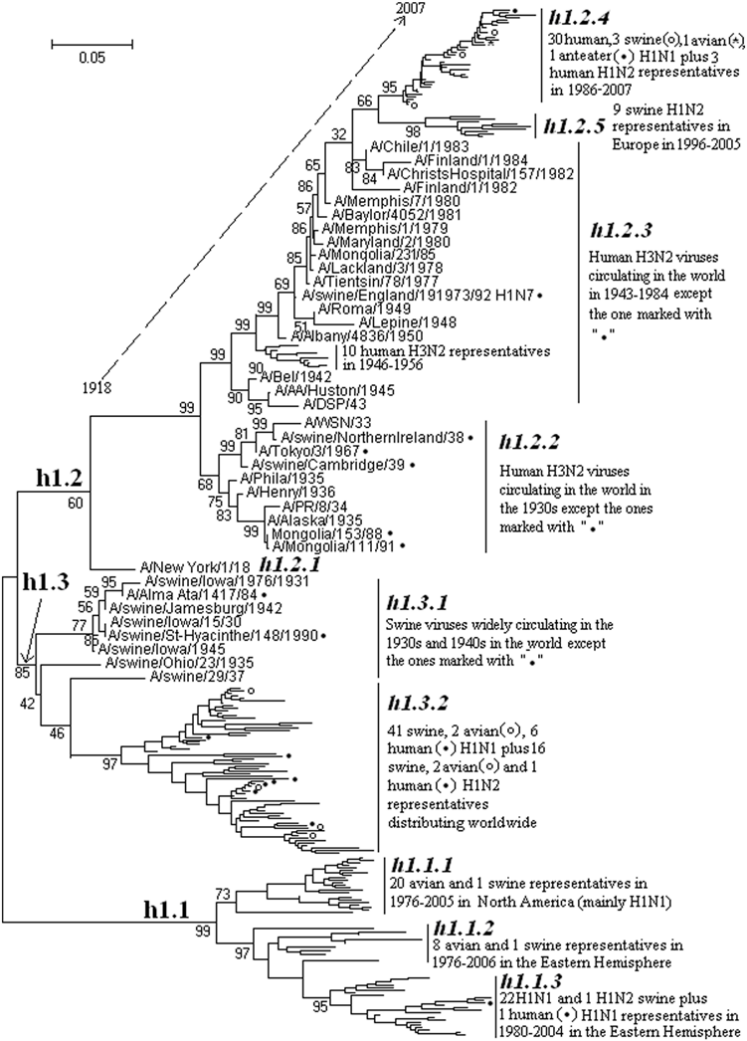
The panorama phylogenetic tree of subtype H1 influenza virus based on the viral HA sequences. The tree suggested that H1 influenza viruses could be divided into 3 lineages largely corresponding to the avian, human and classical swine H1 influenza viruses, respectively. Bootstrap values were given at relevant nodes.

215 (33 avian, 77 human, 104 swine, 1 giant anteater) representatives of the H1 influenza A viruses were selected. Their phylogenetic relationships were given in Figure 3 and consistent with the H1 part in Figure 1. Figure 3 suggested that, from a panorama view, the subtype could be divided into 3 lineages designated as h1.1, h1.2 and h1.3.

Lineage h1.1 corresponded to avian H1 influenza A viruses which could be divided into 3 sublineages, h1.1.1, h1.1.2, h1.1.3. The sublineages of h1.1.1 and h1.1.2 corresponded to avian isolates from North American and the Eastern Hemisphere (including Asia, Europe, Oceania and Africa), respectively. Sublineage h1.1.3 corresponded to some swine isolates in the Eastern Hemisphere which have become the dominant swine H1 influenza A viruses in Europe since the 1980s and appeared in China at least from the 1990s [3], [4]. Figure 3 was consistent with previous studies which indicated that the HA gene of the swine viruses in h1.1.3 originated from the avian viruses in h1.1.2 [5].

Lineage h1.2 corresponded to human H1N1 influenza A viruses isolated from 1918 to 2007. This lineage began with the catastrophic human pandemic in 1918, and then continued with annual epidemics till now except in 1958∼1976 [6]. Interestingly, like the avian lineage h1.1, this human lineage also generated a swine sublineage (h1.2.4) which circulated in Europe at least during 1994∼2005 through genetic reassortment [7], [8]. Beside this sublineage, lineage h1.2 could be divided into four sublineages, h1.2.1∼h1.2.4, largely corresponding the periods 1918, 1930∼1939, 1942∼1985 and 1986∼2007, respectively.

Lineage h1.3 mainly comprised classical swine H1 influenza viruses which circulated in the world from the 1910s until 1979 [4], [9]. Thereafter, the viruses mainly circulated in America and Asia [9], [10]. Sometimes, as demonstrated by Figure 3, humans or birds could be infected by the viruses of this lineage. Moreover, in 1976, more than 200 recruits in New Jersey were infected by this lineage and the abortive epidemic was feared to have pandemic potential [6]. The lineage could be divided into two sublineages, h1.3.1 and h1.3.2, largely corresponding to the period of the 1930s∼1940s and the 1970s∼2000s, respectively.

Only one mammalian isolate, A/swine/England/191973/92(H1N7) was of the NA subtype other than N1 and N2, which indicated it was an isolate resulted from genetic reassortment [11].

The genetic distances between the H1 lineages and those between the H1 sublineages of the same lineage were ranging from 9.3% to 66.0% (mean = 39.3%) and 2.4% to 29.2% (mean = 16.0%), respectively.

### The phylogenetic distribution of H2 subtype influenza viruses

**Figure 4 pone-0005022-g004:**
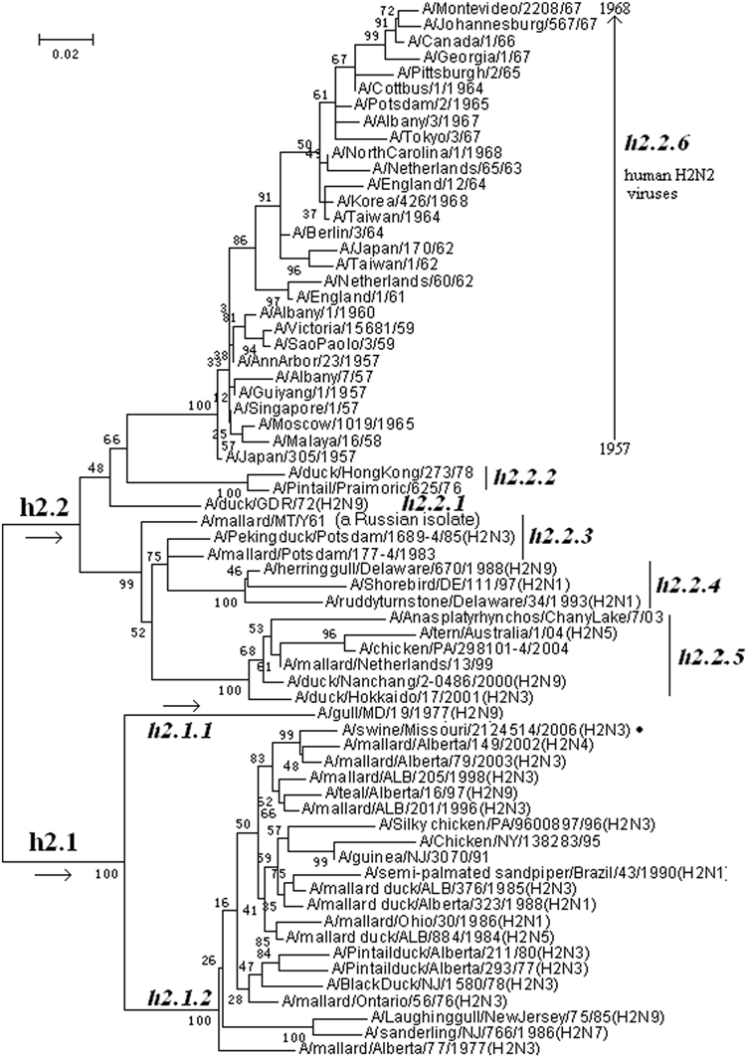
The panorama phylogenetic tree of subtype H2 influenza virus based on the viral HA sequences. The tree suggested that H2 influenza viruses could be divided into 2 lineages, h2.1 and h2.2, comprising the avian viruses isolated from the Western Hemisphere and Eastern Hemisphere, respectively. Human H2N2 influenza viruses circulating in 1957–1968 formed a separate sublineage in lineage h2.2. Few swine H2 influenza viruses (•) were identified within this subtype. Bootstrap values were given at relevant nodes.

66 (36 avian, 1 swine and 29 human) representatives of H2 subtype influenza viruses were selected. Their phylogenetic relationships were given in Figure 4 and consistent with the H2 part in Figure 1.

Figure 4 suggested that, from a panorama view, the subtype could be divided into two lineages designated as h2.1 and h2.2. Lineage h2.1 corresponded to the avian H2 influenza viruses isolated from the Western Hemisphere, while lineage h2.2 was more complicated including human and avian viruses from multiple continents. The two lineages could be further divided into some sublineages. In lineage h2.2, sublineage h2.2.6 corresponded to human H2N2 influenza viruses which circulated in the world during 1957∼1968, and the rest h2.2 sublineages corresponded to the viruses isolated from the Eastern Hemisphere, except sublineages h2.2.4 surprisingly corresponded to the viruses from North America [12].

Figure 4 indicated that the HA gene of human H2N2 influenza viruses were probably originated from avian influenza viruses belonging to lineage h2.1 [13].

The genetic distances between the H2 lineages and those between the H2 sublineages in the same lineage were ranging from 18.0% to 32.5% (mean = 23.3%) and 4.9% to 26.3% (mean = 13.3%), respectively.

### Panorama phylogenetic distribution of H3 subtype influenza viruses

**Figure 5 pone-0005022-g005:**
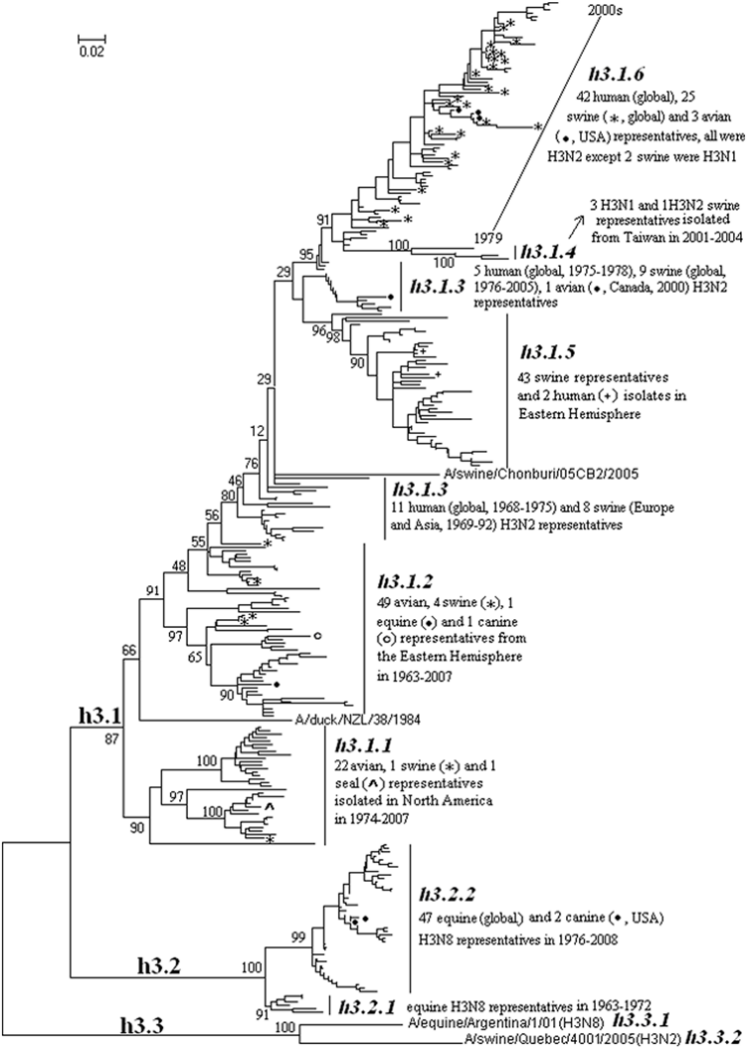
The panorama phylogenetic tree of subtype H3 influenza virus based on the viral HA sequences. Bootstrap values were given at relevant nodes.

301 (98 swine, 84 avian, 60 human, 53 equine, 3 canine, 2 environment and 1 seal) representatives of H3 subtype influenza viruses were selected. Their phylogenetic relationships were given in Figure 5 and consistent with the H3 part in Figure 1. Figure 5 suggested that, from a panorama view, the subtype could be divided into 3 lineages designated as h3.1, h3.2 and h3.3.

Only two representatives, one from a horse in Argentina and the other from a pig in Canada, were identified in lineage h3.3. Considering their genetic distance and distinct background, they were classified into two different sublineages, h3.3.1 and h3.3.2, respectively.

Lineage h3.2 corresponded to equine isolates which were divided into two sublineages, h3.2.1 and h3.2.2, corresponding to the period of 1963∼1972 and 1976∼2007, respectively. The equines viruses in the sublineages have been found caused epidemics in dogs in USA [14], [15].

Lineage h3.1 was rather complicated comprising many human, avian and swine isolates, some of which were intermediate between the distinct sublineages within the lineage, and difficult to be classified thereby as explained by Text S1. The lineage was tentatively classified into 10 sublineages, h3.1.1∼h3.1.10. Sublineage h3.1.1 were all avian viruses isolated in North America during 1974∼2007 except a few viruses causing infections in pigs and seals [16], [17]. Sublineage h3.1.2 consisted of viruses (mainly from birds) isolated from the Eastern Hemisphere. Sublineage h3.1.3 was intermediate between the avian sublineage h3.3.2 and other three sublineages (human sublineage h3.3.6 and swine sublineages h3.3.4 and h3.3.5).

The human viruses in sublineage h3.1.3 sparking the human influenza pandemic in 1968 might derive the HA gene directly from avian sublineage h3.1.2 [18], [19]. The swine sublineage h3.1.4 comprised swine H3N2 viruses isolated in Taiwan in the 2000s, and the swine sublineage h3.1.5 comprised swine H3N2 viruses isolated in 1983∼2007 in the Eastern Hemisphere with some viruses isolated from humans. Sublineage h3.1.6 mainly comprised the human H3N2 viruses causing annual epidemics till now. Swine infections with the viruses in sublineage h3.1.6 were frequently identified [20]–[22].

Because human H3 representative were selected without consideration of isolation places, the 42 human representatives actually represent much more isolates than the 30 swine representatives in h3.1.6 in Figure 5. The 30 swine representatives in h3.1.6 were similar to the human representatives isolated in the same year or earlier, which indicated that pigs might be the victims of the human influenza viruses [1].

Similar to H1 influenza viruses, Figure 5 demonstrated that interspecies transmissions within H3 influenza viruses have been identified in most of the H3 sublineages. Interestingly, some birds were found infected directly with mammalian H3N2 viruses in sublineage h3.1.6 without genetic reassortment [23], [24].

Similar to human H2 influenza viruses, Figure 5 demonstrated that probably human H3 influenza viruses were originated from the avian influenza viruses circulating in the Eastern rather than Western Hemisphere.

In addition, like human H1N1 and H2N2 viruses, human H3N2 influenza viruses changed obviously with time rather than regions (data not shown).

The genetic distances between the H3 lineages and those between the H3 sublineages within the same lineage were ranging from 30.42% to 101.6% (mean = 64.82%) and 4.72% to 16.2% (mean = 10.64%), respectively.

### Panorama phylogenetic distribution of H5 subtype influenza viruses

**Figure 6 pone-0005022-g006:**
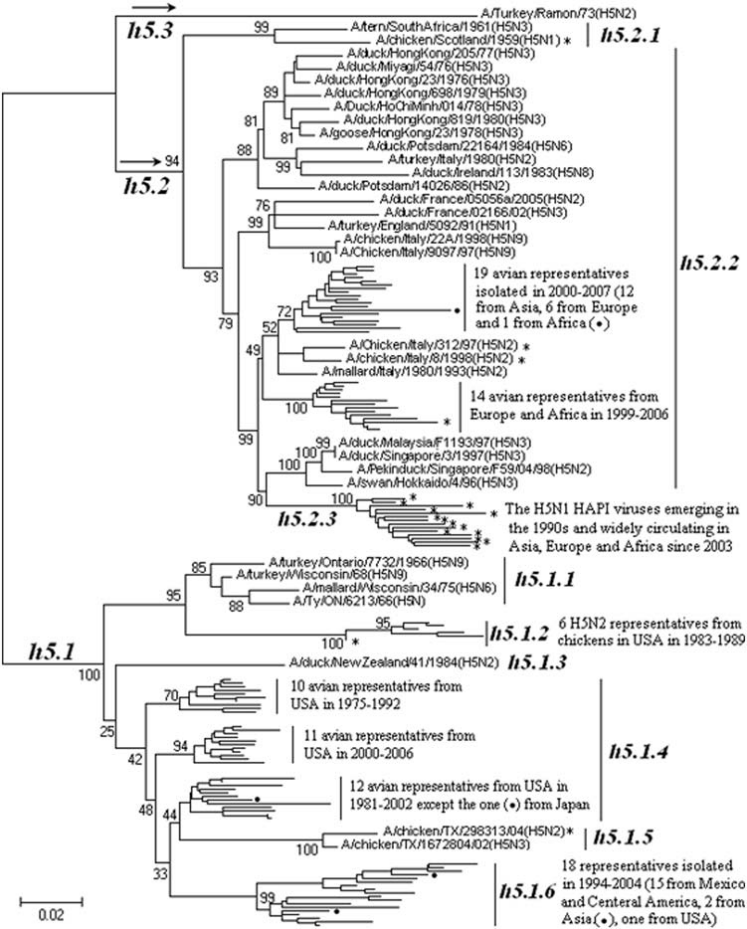
The panorama phylogenetic tree of subtype H5 influenza virus based on the viral HA sequences. The tree suggested that avian H5 influenza viruses isolated from the Western Hemisphere and the Eastern Hemisphere were located in the sublineages h5.1 and h5.2, respectively. The current HPAI H5N1 widely circulating in the Eastern Hemisphere formed a distinct sublineage (h5.2.3) within lineage h5.2 which were partially detailed in Text S2. For space limitation, most representatives within h5.2.3 were not shown in this figure. HPAI viruses were marked with “*”. Bootstrap values were given at relevant nodes.

255 (1 swine, 233 avian, 19 human and 2 cats) representatives of H5 subtype influenza viruses were selected. The phylogenetic relationships of the representatives calculated with their own HA sequences were given in Figure 6 and consistent with the H5 part in Figure 1. Figure 6 suggested that, from a panorama view, the subtype could be divided into 3 lineages designated as h5.1, h5.2 and h5.3.

Only one strain, A/turkey/Ramon/73(H5N2), isolated in Mexico in 1973 was identified in lineage h5.3, while the lineages of h5.1 and h5.2 comprised many avian isolates from the Eastern and Western Hemisphere, respectively. The lineages of h5.1 and h5.2 could be further divided into several sublineages, and some sublineages could be further divided into some clades as demonstrated by the example of sublineage h5.2.3 given in Text S2.

Among lineage h5.1, the viruses within sublineage h5.1.1 were isolated in the early years, and the Mexico isolates were located in a sublineage (h5.1.6) distinct from those of USA isolates.

Among lineage h5.2, viruses within sublineage h5.2.1 were isolated in the early years, and the HPAI H5N1 widely circulating in the Eastern Hemisphere in recent years formed a distinct sublineage, h5.2.3, within lineage h5.2. Its phylogenetic diversity was updated in Text S2, and some new clades, such as 2.1.4, 2.1.5, 2.1.6 and 2.3.5, were added.

Some H5 influenza viruses of different NA subtypes, pathogenicity or from different continents, hosts, or years could be located at the same sublineages, though they were of certain spatial and temporal features (Figure 6).

The genetic distances between the H5 lineages were ranging from 21.19% to 46.29% (mean = 29.48%), and between the H5 sublineages in the same lineage were ranging from 5.27% to 33.64% (mean = 16.56%).

### Panorama phylogenetic distribution of H7, H10 and H15 subtypes of influenza viruses

**Figure 7 pone-0005022-g007:**
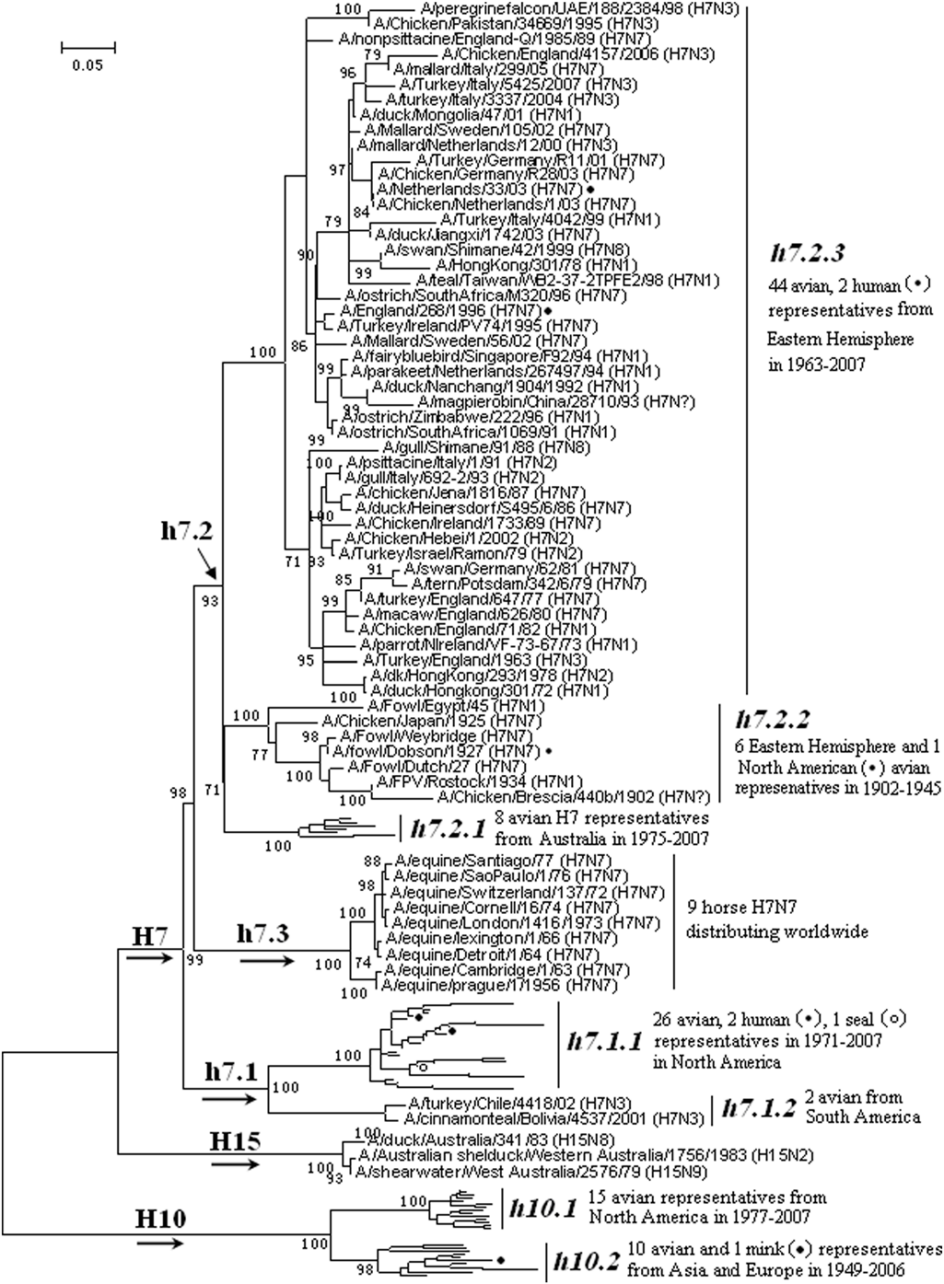
The panorama phylogenetic tree of subtypes H7, H10 and H15 influenza virus based on the viral HA sequences. Bootstrap values were given at relevant nodes.

101 H7 (87 avian, 4 human, 9 horses, 1 seal), 26 H10 (25 avian, 1 mink) and 3 H15 (all avian) representatives were selected. Their phylogenetic relationships were given in Figure 7 and consistent with their counterparts in Figure 1 except that H15 subtype was shown as an outer rather than inner branch of subtype H7.

Figure 7 suggested, from a panorama view, that subtype H10 could be divided into 2 lineages, h10.1 and h10.2, corresponding to the avian H10 influenza viruses isolated in the Western Hemisphere and the Eastern Hemisphere, respectively.

From a panorama view, Figure 7 indicated that subtype H7 could be divided into 3 lineages, h7.1, h7.2 and h7.3. Lineage h7.3 corresponded to the equine H7N7 viruses which have not been found since the end of the 1970s [26]. Lineage h7.1 and h7.2 largely corresponded to the avian H7 influenza viruses isolated in the Western Hemisphere and the Eastern Hemisphere, respectively. Lineage h7.1 was further divided into two sublineages, h7.1.1 and h7.1.2, corresponded to the avian H7 influenza viruses isolated in North and South America, respectively.

Figure 7 confirmed that the H7 influenza viruses isolated from humans and seals were all of avian sources [27]–[30].

The genetic distances between the H7 lineages and those between the H7 sublineages in the same lineage were ranging from 27.4% to 52.2% (mean = 42.2%) and 5.3% to 37.2% (mean = 24.3%), respectively. The genetic distances between the H10 lineages were ranging from 23.9% to 31.9% (mean = 27.2%).

### Panorama phylogenetic distribution of H9 subtype influenza viruses

**Figure 8 pone-0005022-g008:**
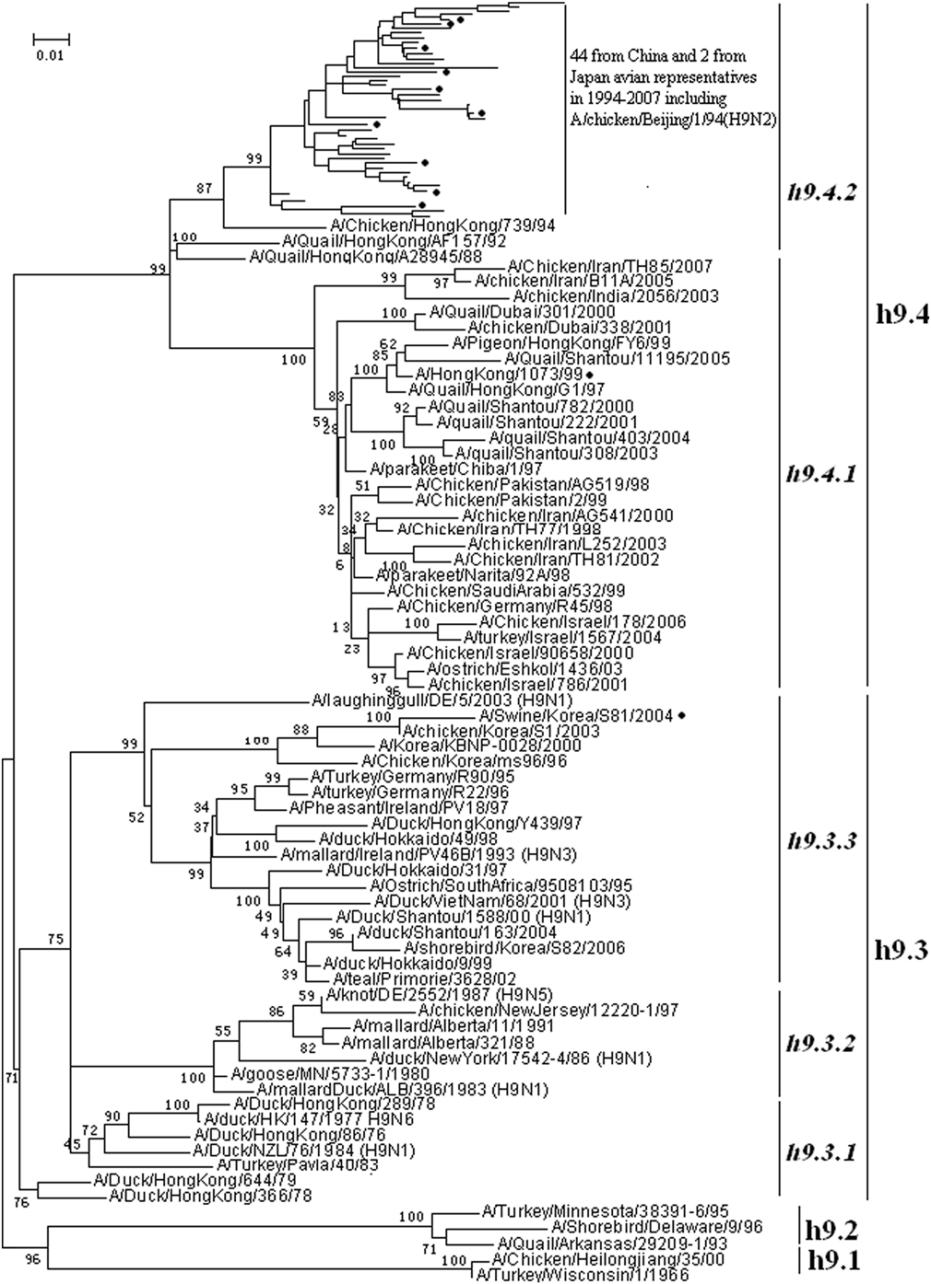
The panorama phylogenetic tree of subtype H9 influenza virus based on the viral HA sequences. The strains marked with “•” were isolated from mammals. Bootstrap values were given at relevant nodes.

115 (102 avian, 8 swine, 5 human) representatives of H9 subtype influenza viruses were selected. Their phylogenetic relationships based on their HA sequences were given in Figure 8, which were consistent with the H9 part in Figure 1 and previous studies on the diversity of H9 influenza viruses [31]–[33]. Figure 8 suggested that, from a panorama view, the subtype could be divided into four lineages designated as h9.1∼h9.4. Lineages h9.3 and h9.4 could be further divided into some sublineages (Figure 8).

Lineage h9.1 corresponded to the strain, A/Ty/Wisconsin/1/1966, isolated in 1966 in North America. The lineages also comprised an odd strain, A//Chicken/Heilongjiang/35/00, isolated in China in 2000 with HA 99.9% homogenous to A/Ty/Wisconsin/1/1966.

Lineage h9.2 comprised a few avian H9 influenza viruses isolated in the 1990s in North America. Lineage h9.3 was a little more complicated. It mainly comprised avian H9 viruses isolated from the Eastern Hemisphere and also harbored some avian H9 influenza viruses isolated from North America. Lineage h9.4 comprised avian H9 influenza viruses from the Eastern Hemisphere. Most avian H9N2 viruses isolated in China in recent years were located in the sublineage h9.4.2. Human infections with the avian viruses in h9.4 have been identified and of public concerns [36].

The genetic distances between the H9 lineages and those between the H9 sublineages in the same lineage were ranging from 7.2% to 31.5% (mean = 23.2%) and 5.9% to 21.8% (mean = 12.0%), respectively.

### Panorama phylogenetic distribution of other HA subtypes of influenza viruses

**Figure 9:**
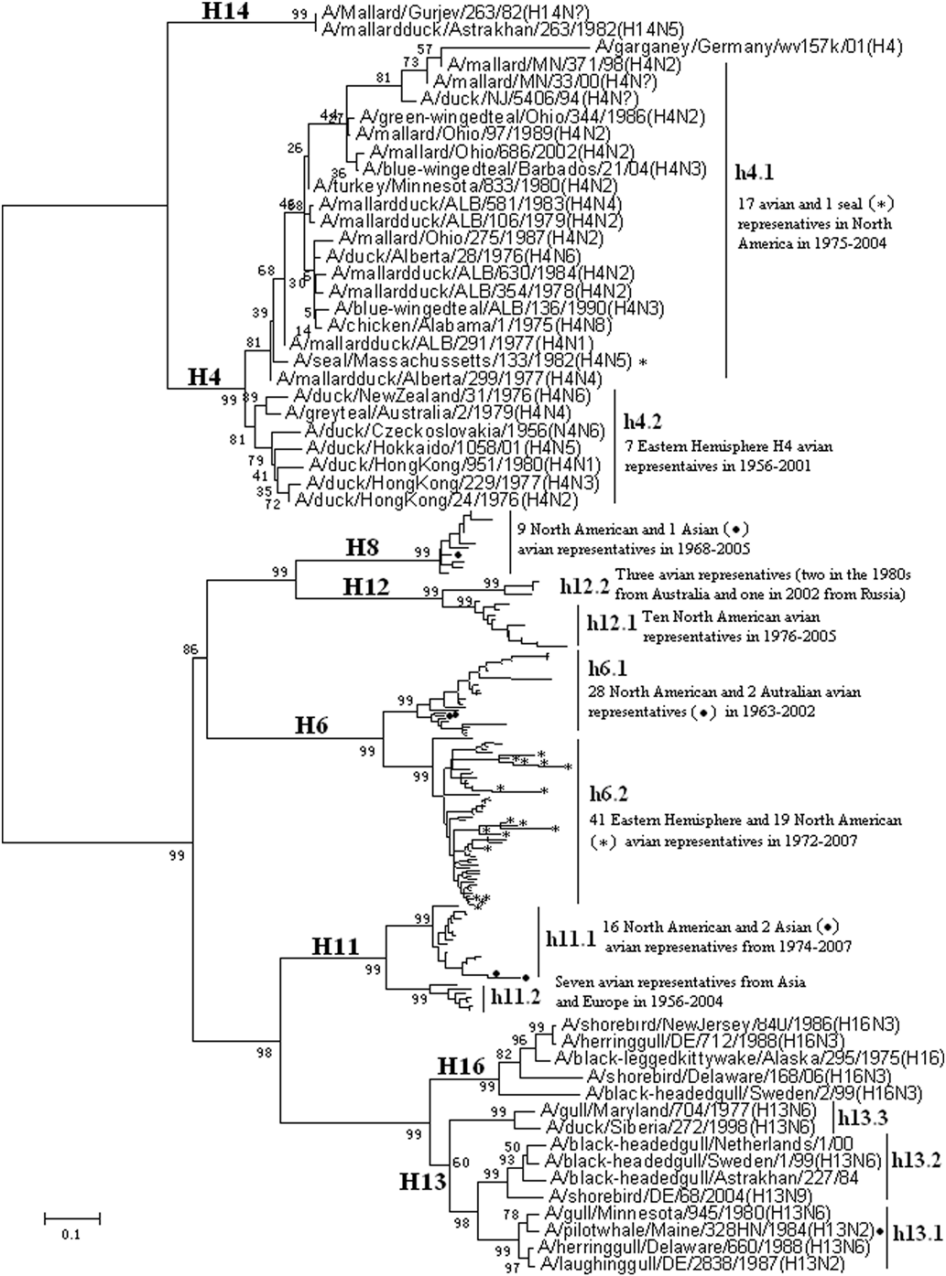
The panorama phylogenetic tree of subtypes H4, H6, H8, H11, H12, H13 and H16 based on the viral HA sequences. Bootstrap values were given at relevant nodes.

182 representatives of other HA subtypes were selected from 635 (374 H6, 132 H4, 12 H8, 65 H11, 24 H12, 15 H13, 3 H14 and 10 H16) influenza viruses. Their phylogenetic relationships based on their HA sequences were given in Figure 9 and largely consistent with their counterparts in Figure 1.

From Figure 9, subtypes H8, H14 and H16 could not be divided into some lineages due to too few isolates available, and subtypes H4, H11 and H12 all were divided into two lineages largely corresponding to avian isolates from the Western and Eastern Hemisphere, respectively. Subtype H6 was also divided into the two lineages, but not that strictly corresponding to the two hemispheres. Subtype H13 could be divided into three lineages without obvious temporal or geographical features.

The genetic distances between the lineages within subtypes H4, H6, H11, H12 and H13 were ranging from 11.3% to 27.5% (mean = 22.7%), 25.8% to 51.1% (mean = 37.9%), 25.2% to 30.8% (mean = 27.9%), 24.6% to 34.2% (mean = 27.6%) and 11.4% to 36.6% (mean = 28.2%), respectively.

### Panorama phylogenetic distribution of N1 subtype influenza viruses

**Figure 10:**
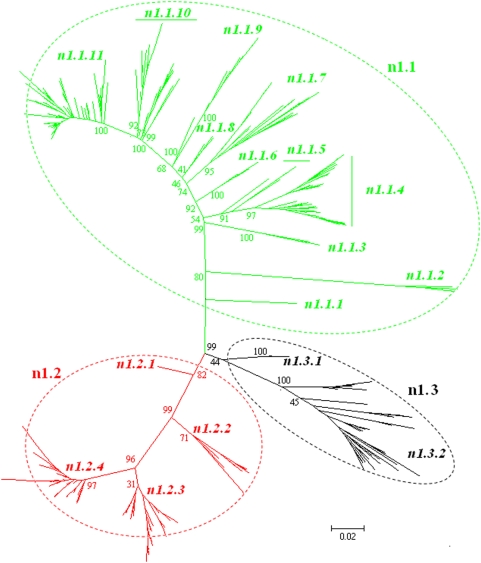
The panorama diversity of N1 influenza viruses which could be divided into 3 lineages, n1.1, n1.2 and n1.3, largely corresponding to avian, human and classical swine N1 influenza viruses. Bootstrap values were given at relevant nodes.

441 representatives of N1 subtype influenza viruses were selected. Their phylogenetic relationships were given in Figure 10 and largely consistent with the N1 part in Figure 2.

Figure 10 was consistent with a previous report on panorama of N1 influenza viruses [34]. It classified N1 influenza viruses into 3 lineages, n1.1, n1.2 and n1.3, largely corresponding to avian, human and classical swine N1 influenza virus. Each of them was further divided into several sublineages which were of certain distinct temporal, geographical or host features (Table S2). Some swine isolates formed a distinct sublineage (n1.1.7) within the avian lineage n1.1, and their HA gene sequences belonged to sublineage h1.1.3 (Figure 3). The current H5N1 HPAI viruses circulating in the Eastern Hemisphere were located in the sublineage n1.1.11.

The genetic distances between the N1 lineages and those between the N1 sublineages in the same lineage were ranging from 7.3% to 36.2% (mean = 22.8%) and 1.7% to 29.9% (mean = 15.3%), respectively.

### Panorama phylogenetic distribution of N2 subtype influenza viruses

**Figure 11 pone-0005022-g011:**
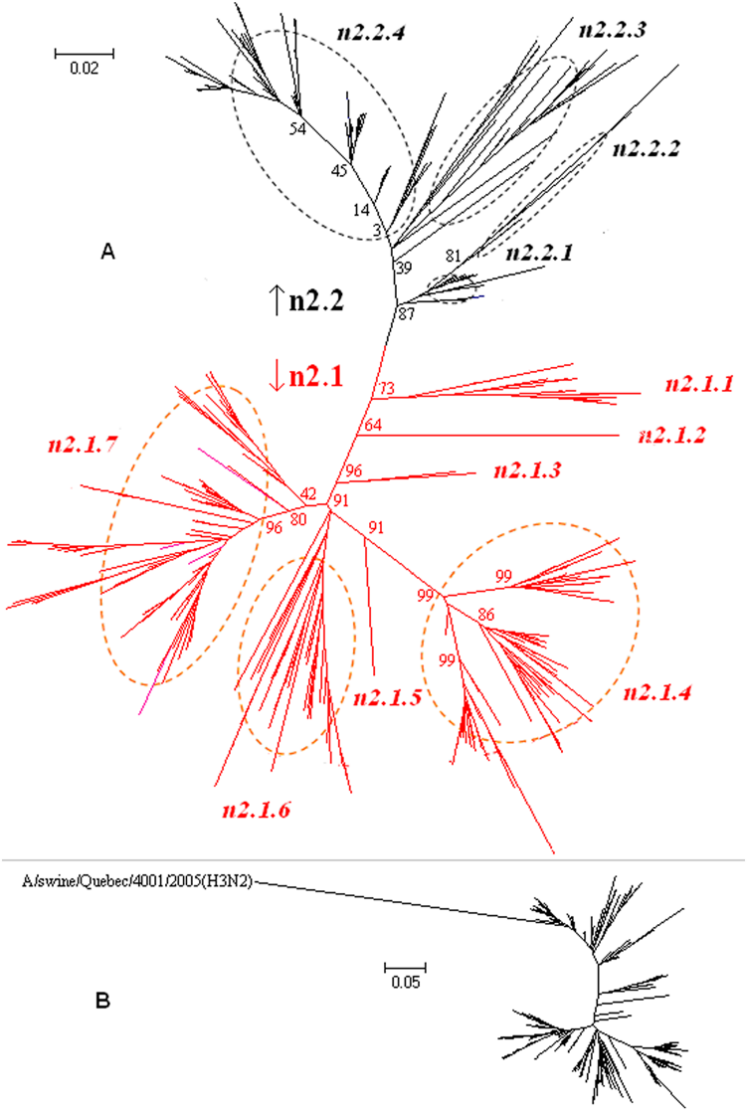
The panorama phylogenetic diversity and distribution of N2 influenza viruses without (A) and with (B) the isolate A/swine/Quebec/4001/2005(H3N2). Bootstrap values were given at relevant nodes.

382 representatives of N2 subtype influenza viruses were selected. Their phylogenetic relationships were given in Figure 11 and largely consistent with the N2 part in Figure 2.

Figure 11 classified N2 influenza viruses into 2 lineages, n2.1 and n2.2, largely corresponding to avian and mammalian N2 influenza viruses (Figure 11A). Each of them could be divided into several sublineages which were of certain distinct temporal, geographical or host features (Table S2). Some sublineages, like n2.2.3, n2.2.4 and n2.1.4, could be further divided into some clades. A/swine/Quebec/4001/2005(H3N2) was a special isolate within N2 subtype and could be assigned as another lineage within N2 subtype (Figure 11B).

The genetic distances between the N2 lineages and those between the N2 sublineages in the same lineage were ranging from 25.8% to 51.1% (mean = 37.9%) and 4.9% to 27.9% (mean = 15.1%), respectively, without consideration of the special isolate A/swine/Quebec/4001/2005(H3N2). The genetic distances between A/swine/Quebec/4001/2005(H3N2) and other N2 influenza viruses were ranging from 38.4%∼65.5% (mean = 54.3%).

### Panorama phylogenetic distribution of N3 subtype influenza viruses

**Figure 12 pone-0005022-g012:**
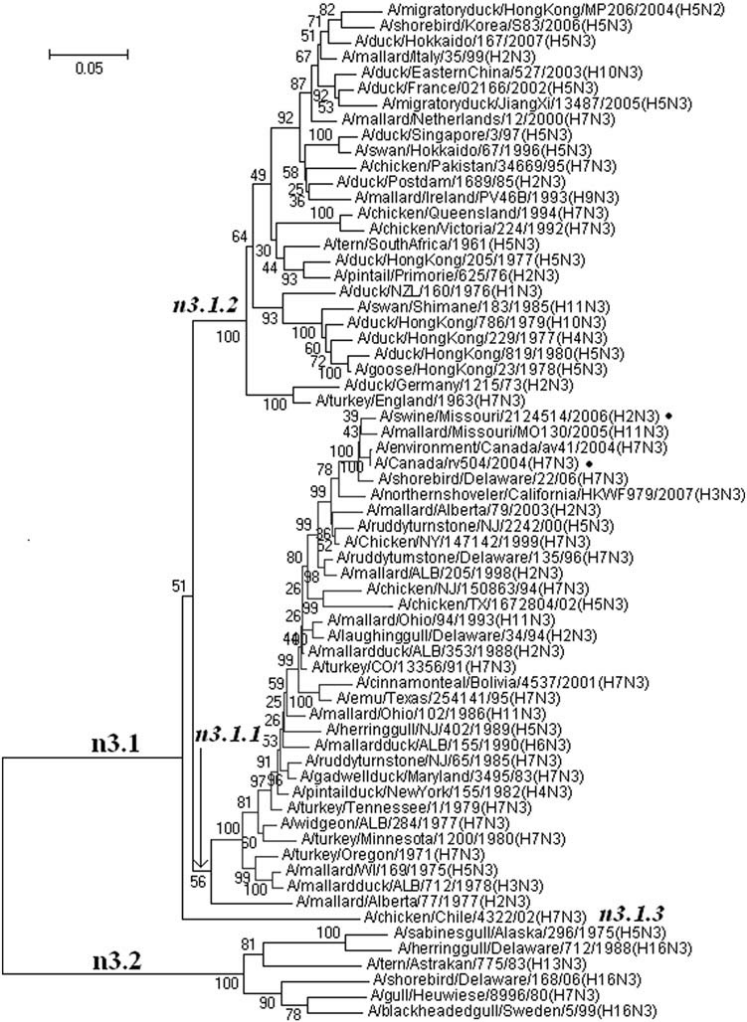
The panorama diversity of subtype N3 influenza viruses which could be divided into two lineages. Lineage n3.1 was further divided into 3 sublineages. Some viruses in sublineage n3.1.2 isolated from humans or pigs were marked with “•”. Bootstrap values were given at relevant nodes.

65 representatives of N3 subtype influenza viruses were selected. Their phylogenetic relationships were given in Figure 12 and largely consistent with the N3 part in Figure 2.

Figure 12 classified N3 influenza viruses into 2 lineages, n3.1 and n3.2. Lineage n3.1 was further divided into 3 sublineages. Sublineage n3.2 did not show any obvious geographical features. Some viruses in sublineage n3.1.1 were isolated from humans or pigs.

The genetic distances between the N3 lineages were ranging from 40.8% to 52.3% (mean = 45.2%).

### Panorama phylogenetic distribution of subtypes N4, N5 and N8 influenza viruses

**Figure 13 pone-0005022-g013:**
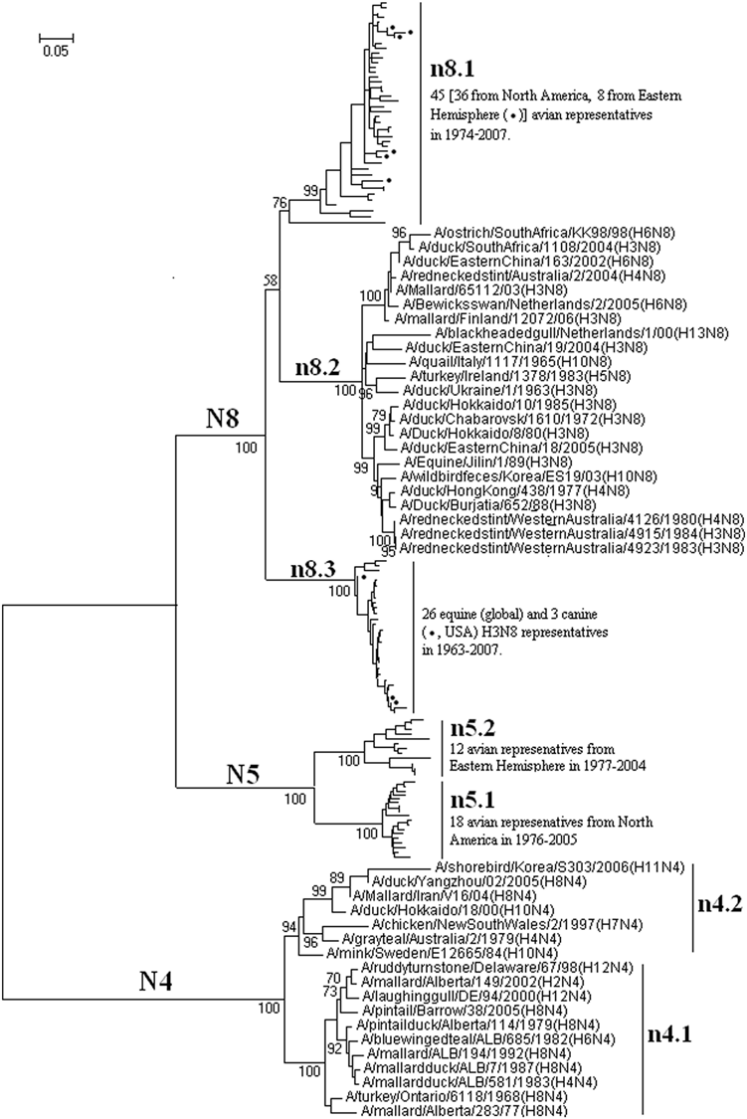
The panorama diversity of subtypes N4, N5 and N8 influenza viruses. Avian influenza viruses of these subtypes all could be classified into 2 lineages corresponding to the Western and Eastern Hemisphere, respectively, with some exceptions in sublineage n8.1. The equine H3N8 influenza viruses formed a separate lineage (n8.3) within subtype N8. Bootstrap values were given at relevant nodes.

144 representatives of subtypes N4, N5 and N8 influenza viruses were selected. Their phylogenetic relationships were given in Figure 13 and largely consistent with their counterparts in Figure 2.

Figure 13 demonstrated that avian influenza viruses of these three subtypes all could be classified into 2 lineages corresponding to the Western Hemisphere (n4.1, n5.1 and n8.1) and Eastern Hemisphere (n4.2, n5.2 and n8.2), respectively, with some exceptions in sublineage n8.1. The equine N8 influenza viruses formed a separate lineage within subtype N8.

The genetic distances between the N4 lineages, the N5 lineages and the N8 lineages were ranging from 17% to 27.7% (mean = 21.9%), 24.9% to 34.5% (mean = 28.7%) and 20.5.9% to 46.8.9% (mean = 34.8%), respectively.

### Panorama phylogenetic distribution of subtypes N6, N7 and N9 influenza viruses

**Figure 14:**
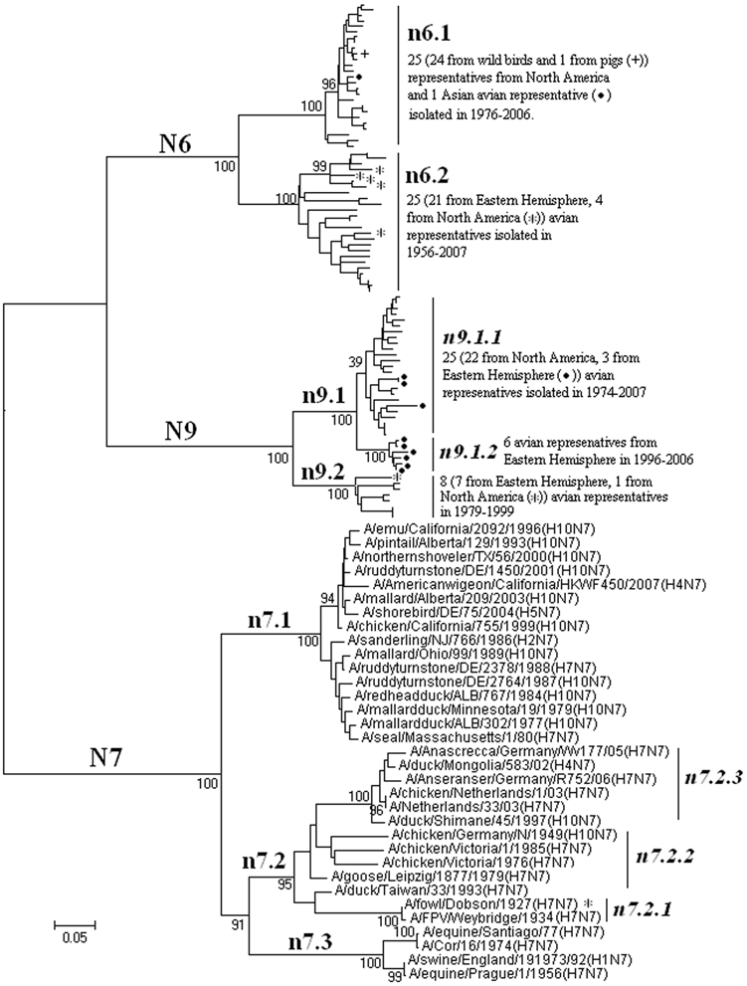
The panorama diversity of subtypes N6, N7 and N9 influenza viruses. Avian influenza viruses of these subtypes all could be classified into 2 lineages corresponding to the Western and Eastern Hemisphere, respectively, with some exceptions marked with “*” or “•”. The equine N7 influenza viruses formed a separate lineage (n7.3) within subtype N7. Bootstrap values were given at relevant nodes.

123 representatives of subtypes N6, N7 and N9 influenza viruses were selected. Their phylogenetic relationships were given in Figure 14 and largely consistent with their counterparts in Figure 2.

Figure 14 demonstrated that avian influenza viruses of subtypes N6, N7 and N9 all could be classified into 2 lineages corresponding to the Western Hemisphere (n6.1, n7.1 and n9.1) and Eastern Hemisphere (n6.2, n7.2 and n9.2), respectively, with some exceptions in lineages n6.1, n6.2, n9.1, n9.2 and n7.2. Lineage n9.1 was further divided into 2 sublineages which were of different geographical distribution, and lineage n7.2 was further divided into 3 sublineages with difference in temporal distribution (Figure 14 and Table S2).

The genetic distances between the N6 lineages, the N7 lineages and the N9 lineages were ranging from 26.6% to 35.1% (mean = 30.4%), 32.3% to 42.2% (mean = 37.8%) and 23.1% to 29% (mean = 25.4%), respectively.

## Discussion

The topologies of the phylogenetic trees described here were all consistent with, though much more covering than, previous reports [1]–[39]. During the analysis of the sequences, we found that the topology of phylogenetic trees changed little if calculated with different software packages like MEGA, PHYLIP or PAUP, or calculated with different methods like neighboring-joining or maximum parsimony (data not shown). But it could change greatly using different parameters. In this study, we set the nucleotide sequences changing in Kimura-2-Parameter gamma model because we found that transitional substitution rates were much higher than transversional ones in the viral evolution, and substitution rates were significantly different among sites. In addition, it is important to treat gaps by pairwise-deletion rather than complete-deletion, i.e. ignoring only those gaps that are involved in the comparison of a pair of sequences because random insertions or deletions were frequent in the evolution of the two viral genes especially among different subtypes.

During the preliminary analysis of the sequences, we found that the trees might change greatly if some short sequences were involved. Therefore, in this study, sequences shorter than a limitation were excluded.

It is possible that the panorama views reported here reflected only a part of the reality. First, few isolates have been reported from South America and Africa, and influenza viruses of marine mammals also remain largely unknown. Second, it is possible that a few sequences reported to GenBank were with some errors which could distort the phylogenetic “maps” described here.

Nevertheless, the panorama maps help us to recognize influenza A viruses from panorama views, and provided a framework for to generalize the history and explore the future of the viral circulation in multiple kinds of hosts. For example, the pandemic of human H3N2 virus in 1968 [19], the severe outbreak of equine influenza with an avian-like H3N8 virus in China in 1989 [35], and the crisis of H5N1 HPAI in Hong Kong in 1997 [36], all could be marked in the maps. As for the future, it is easier with the guide of the panorama maps to identify whether an isolate is special or odd in epidemiology (like A/equine/Argentina/1/01(H3N8) in lineage h3.3.1 in Figure 5), and whether an isolate originated from genetic reassortment, like the aforementioned A/swine/England/191973/92 (H1N7) [11].

The panorama maps gave us some new information to recognize influenza A virus. First, they demonstrated that H13 and H16 were very close in genetics, and so did subtype H7 and H15. The maps also indicated that some subtypes like H3, H5, H7 and H9 might have evolved into some subtypes during the evolution in the past decades, which, however, should be confirmed by serological tests. Second, avian influenza viruses of each subtype were usually classified into Eurasian and North American lineages in the past presumably owing to confinement of birds to the distinct flyways of each hemisphere [37]–[39], and here the two lineages were confirmed within H1, H3, H5, H7, H8, H10, N4, N5, N6 and N8 subtypes, but their geographical distribution should be enlarged to the Eastern (Asia, Oceania, Europe and Africa) and Western Hemisphere (North and South America), respectively. Moreover, the avian influenza viruses within subtypes H2, H4, H6, H9, H11, H13, N1, N2, N3, N7 and N9 were more complicated than these two lineages. For example, avian H9 influenza viruses should be divided into 4 lineages, two for each hemisphere, and some avian viruses isolated from the Western Hemisphere was located in one sublineage (h9.3.2) of the lineage h9.3 corresponding to the Eastern Hemisphere.

The panorama maps demonstrated that swine H1 and H3 viruses were of high diversity, and swine infections with avian or human H1 and H3 influenza viruses as well as human or avian infections with swine H1 and H3 viruses were rather frequent. Therefore, the maps offered us a new dimension to recognize that pigs may play a important role in the ecology and epidemiology of H1 and H3 influenza viruses.

The panorama maps also demonstrated that unlike swine and avian influenza viruses, human influenza viruses demonstrated obvious temporal but little geographical difference, and they have diverged into few clades during the past decades. These suggested that human viruses update rapidly and they were maintained almost exclusively by humans themselves. Similar features were also found in equine influenza viruses. The features indicated that the circulation of human and equine influenza A viruses was vulnerable, which was supported by the stop of the circulation of human H2N2 and equine H7N7 influenza viruses [1].

The phylogenetic distribution reported here suggests that, from a whole view, there is a little possibility for an avian influenza virus to infect humans or other mammals, but little possibility for a mammalian influenza virus to infect birds, though some bird infections with swine H1N1 viruses in sublineage h1.3.2 and H3N2 viruses in sublineage h3.1.6 have been found. Therefore, the panorama analysis supported that influenza viruses lost their adaptability in birds while they have become adapted to mammalian hosts [1].

The panorama phylogenetic trees are useful for detection of the viruses. For example, the primers of the current China national standard RT-PCR for the detection of H7 subtype avian influenza viruses, 5-AATGCACARGGAGGAGGAACT-3 (upper) and 5-TGAYGCCCCGAAGCTAAACCA-3 (down), are specific to lineage h7.2 rather than h7.3, while the later corresponds to the avian H7 influenza viruses isolated in the Eastern Hemisphere and should be more risky to China according to their phylogenetic distribution. Therefore, the primers should be revised to those corresponding to lineage h7.3 or both h7.2 and h7.3. For another example, a few pairs of primers may be required to detect swine H1 or H3 influenza viruses using RT-PCR as they are of high diversity demonstrated by the panorama views. In addition, the representative sequences selected by this report could give a window to identify the specific and conserved regions in the viral HA and NA sequences for the primer design.

Classification and designation of the lineages and sublineages within influenza A virus were essential for the studies of the viral evolution, ecology and epidemiology [1, 4–44]. In this report, 60 lineages and 83 sublineages within influenza A virus were identified, and all of them were supported by the topology of the phylogenetic trees and with high bootstrap values (>70) at relevant nodes except lineage n1.2 and sublineages h2.2.3, n1.1.3, n1.3.8, n2.1.3, n2.1.4, n2.2.7, n3.1.2 due to the existence of intermediate isolates as elucidated by Text S1 and demonstrated by the exploration of the origin of two lineages of influenza B viruses reported previously [40].

In this report, we tentatively proposed a kind of nomenclature for the lineages and sublineages within influenza A virus. It is informative and specific as it begins with the subtype information. Because only two hierarchies are involved in the nomenclature for simplicity, some sublineages were probably evolved from other sublineages, like sublineage h5.2.3 was possibly evolved from sublineage h5.2.2 which, in turn, was possibly evolved from sublineage h5.2.1 at least in terms of the HA gene (Figure 6). Meanwhile, some sublineage could be further divided into some distinct clades as shown by the example given in Text S2.

The nomenclature reported here covers nearly all known influenza A viruses, and it is easy to be expanded to meet the future evolution of the viruses. For example, a new lineage, n2.3, could be assigned to the special isolate in Figure 11B, and some new sublineages could be assigned to the viruses, A/duck/NZL/38/1984 and A/swine/Chonburi/05CB2/2005 in Figure 5. Moreover, the WHO/FAO/OIE classification and designations of the clades and subclades within the sublineage h5.2.3 (Text S2) also provided a kind of expansion of the nomenclature of the lineages and sublineages reported here.

The nomenclature is easy to remember as it is of certain orders as given in Methods. For example, lineages h1.1, n1.1 and n2.1 all corresponded to avian influenza viruses, and lineages h2.1, h3.1, h4.1, h5.1, h6.1, h7.1, h10.1, h11.1 and h12.1 all largely corresponded to the avian influenza viruses isolated from the Western Hemisphere.

If this nomenclature is widely accepted, it should facilitate international communication on the evolution, ecology and epidemiology of influenza A viruses through the unification of previous miscellaneous nomenclatures which were not only ambiguous or misleading in some cases, but also covered only a part of the diversity of influenza A viruses. For example, the previous designation “swine human-like H1 viruses” was ambiguous as the swine viruses within the human H1 lineage belonged to multiple sublineages (Figure 3), and the previous designation “Eurasian lineage of H2 influenza virus” actually comprised viruses from Australia and Africa and even some from North America (Figure 4). Moreover, a substantial part of subtypes H3, H5, H9, N3, etc., have not or could not be covered by previous nomenclatures.

## Methods

### Selection and primary analysis of representative sequences

All the around 15000 HA and 8000 NA sequences of influenza A viruses available in GenBank till July 11, 2008 were analyzed firstly through the web servers of Influenza Virus Resource in NCBI [41], to exclude the sequences of the same viruses, the viruses with unclear background, or manipulated materials and the sequences shorter than 500 bp or with sequencing errors like the ones with GenBank accession numbers AX822028, AX822031, AX822045. The rest sequences were then utilized for further analysis (assortment, alignment and tree-building) through the web servers of Influenza Virus Resource to elucidate their host and subtype distributions and identify the number of basic amino acid residues at the cleavage site in the HA sequences.

The representative sequences were selected by randomly choosing one from those which were similar to each other (approximately genetic distances <2.1%), isolated from the similar host (avian or a species of mammals) in the same year and in the same country. Since human H1N1, H2N2 and H3N2 subtypes viruses did not shown obvious geographical bias through primary analysis and they were of large numbers, their representatives were selected by randomly choosing one from those which were similar to each other and isolated in the same year no matter where they were isolated.

### Calculation of genetic distances

Genetic distances among the representative sequences were calculated using the model of Kimura-2-Parameter using the software MEGA 4.0 [42]. The substitution rates among sites were set in Gamma distribution. The Gamma parameter was set as 1.0. Gaps were treated by pairwise-deletion, i.e. ignoring only those gaps that are involved in the comparison of a pair of sequences. The genetic distances between lineages and those between sublineages were calculated through the comparison of the selected representative sequences.

### Phylogenetic analysis

Representative sequences were aligned using the software of Clustal X [43], and their phylogenetic relationship was analyzed using the software MEGA 4.0 with neighboring-joining method and the same parameters for the genetic distance calculation [42]. with neighboring-joining method [42] using the same parameters for the genetic distance calculation. Bootstrap values were calculated out of 1000 replicates. All the phylogenetic trees were calculated by two separate groups to avoid manual errors.

### Classification of lineages and sublineages

Lineages and sublineages were classified according to genetic distances and topology of the phylogenetic trees. Distributions of the viruses in hosts, regions and time were also considered in the classification. Lineage designation began with the subtype name which was followed by a point and a number (e.g. n2.1), and sublineage designation began with its lineage designation which was followed by a point and a number (e.g. n2.1.1). The numbers within the designations of the lineages and sublineages, if possible, were in the order of avian, human, swine, equine followed by others in terms of hosts, and in the order of the Western Hemisphere followed by Eastern Hemisphere in geography, and in the order from the past to nowadays in isolation time.

## Supporting Information

Table S1 Distribution of the lineages and sublineages within subtypes H1–H16 influenza viruses
(0.20 MB DOC)

Table S2 Distribution of the lineages and sublineages within subtypes N1–N9 influenza viruses
(0.15 MB DOC)

Text S1 The impact of intermediate strains on phylogenetic classification
(0.04 MB DOC)

Text S2 Panorama phylogenetic distribution of the viruses in sublineage h5.2.3 in 2005∼2008.
(0.08 MB DOC)
