# Supplementary material for: Panorama Phylogenetic Diversity and Distribution of Type A Influenza Virus
Source: PLoS One. 2009 Mar 27;4(3):e5022. doi: 10.1371/journal.pone.0005022 (PMC2658884; doi:10.1371/journal.pone.0005022)
Supplement: Table S1 — Distribution of the lineages and sublineages within subtypes H1–H16 influenza viruses (0.20 MB DOC) [file pone.0005022.s001.doc]

**Supporting Information 1:**

**The hosts, isolation periods, isolation places and NA subtypes of the lineages and sublineages within subtypes H1~H16.**

| **Lineage/ sublineage** | **A representative isolate** | **Host** | **Isolation period** | **Isolation place** | **NA subtype** | **Other information** |
| --- | --- | --- | --- | --- | --- | --- |
| h1.1 |  | Birds and pigs | 1976-2006 | Global | Major: N1 |  |
| h1.1.1 | A/duck/Alberta/35/76(H1N1) | Major: birds  Minor: pigs | 1976-2005 | North America | Major: N1 |  |
| h1.1.2 | A/duck/Miyagi/66/77(H1N1) | Major: birds  Minor: pigs | 1976-2006 | Eastern Hemisphere | Major: N1 |  |
| h1.1.3 | A/swine/Netherlands/3/80(H1N1) | Most: Pigs  One: human | 1980-2004 | Eastern Hemisphere | Major: N1 Minor: N2 | This sublineage was evolved from the avian viruses in h1.1.2. |
| h1.2 |  | Major: humans | 1918-2007 | Global | Major: N1  Minor: N2 | Some exceptions were isolated from pigs or birds. |
| h1.2.1 | A/New York/1/18 | Humans | 1918 | Global | N1 | The virus caused the human pandemic in 1918-1919. |
| h1.2.2 | A/PR/8/34 | Major: Humans  Minor: Pigs | Major: 1930s | Global | N1 | Some viruses from human vaccines were isolated in the 1980s and 1990s. |
| h1.2.3 | A/Bel/1942 | Most: humans | 1942-1985 | Global | N1 | An exception was isolated in the 1990s in England from a pig. |
| h1.2.4 | A/Taiwan/1/1986(H1N1) | Major: humans | 1986-2007 | Global | Major: N1  Minor: N2 | Some exceptions were isolated from pigs and anteaters. |
| h1.2.5 | A/swine/England/690421/95 | Pigs | 1995-2005 | Western Europe | N2 | This sublineage was generated through genetic reassortment. |
| h1.3 |  | Major: pigs | 1930s-2000s | Global | Major: N1  Minor: N2 | This lineage was equal to previous “classical swine H1 lineage”. |
| h1.3.1 | A/swine/lowa/15/30 | Most: pigs | Major: 1930s-1940s | Global | N1 |  |
| h1.3.2 | A/swine/Wisconsin/1/1957(H1N1) | Most: pigs  Some: humans and birds | 1957-2000s | Global | Major: N1  Minor: N2 | This sublineage caused an abortive epidemic in humans in USA in 1976. |
| *h2.1* |  | *Most: birds* | *1976-2006* | *Western Hemisphere* | *Multiple* |  |
| *h2.1.1* | *A/gull/MD/19/1977(H2N9)* | *Birds* | *1977* | *Western Hemisphere* | *N9* | *Only one representative was available.* |
| *h2.1.2* | *A/mallard/Ontario/56/76(H2N3)* | *Most: birds* | *1976-2006* | *Western Hemisphere* | *Multiple* | *An exception was isolated from a pig.* |
| *h2.2* |  | *Birds and humans* | *1957-2004* | *Global* | *Major: N2* |  |
| *h2.2.1* | *A/duck/GDR/72* | *Birds* | *1972* | *Germany* | *N9* | *Few isolates were available.* |
| *h2.2.2* | *A/Pintail/Praimoric/625/1976* | *Birds* | *1976,1978* | *Eastern Hemisphere* | *N2* | *Few isolates were available.* |
| *h2.2.3* | *A/mallard/Postam/177-4/1983(H2N2)* | *Birds* | *1961-1985* | *Germany, Russia* | *N2, N3* | *Few isolates were available.* |
| *h2.2.4* | *A/Shorebird/DE/111/97(H2N1)* | *Birds* | *1988-1997* | *USA* | *N1, N9* | *Few isolates were available.* |
| *h2.2.5* | *A/mallard/Netherlands/13/99* | *Birds* | *1999-2004* | *Most: Eastern Hemisphere* | *Multiple* | *One exception was from North America.* |
| *h2.2.6* | *A/Guiyang/1/1957* | *Humans* | *1957-1968* | *Global* | *N2* |  |
| h3.1 |  | Humans, birds and pigs | 1963-2008 | Global | multiple |  |
| h3.1.1 | A/mallard/Ohio/181/1986(H3N1) | Most: birds | 1974-2007 | North America | Multiple | Two exceptions were from a pig and a seal, respectively. |
| h3.1.2 | A/duck/HongKong/7/1975(H3N2) | Major: birds | 1963-2007 | Eastern Hemisphere | Multiple | Some exceptions were from pigs, horses or dogs. |
| h3.1.3 | A/NorthernTerritory/60/1968(H3N2) | Major: humans and pigs | 1968-2005 | Global | N2 | This is an intermediate sublineage. One exception was avian. |
| h3.1.4 | A/swine/Taiwan/0408/2004(H3N1) | Pigs | 2001-2004 | Taiwan | N1, N2 | Few isolates were available. |
| h3.1.5 | A/swine/IDT/Re220/92(H3N2) | Major: pigs | 1983-2006 | Eastern Hemisphere | Most: N2  Minor: N1 | Two exceptions were from humans. |
| h3.1.6 | A/SouthAfrica/56/98(H3N2) | Major: humans  Minor: pigs | 1979-2008 | Global | Major: N2  Minor: N1 | Three exceptions were avian. |
| h3.2 |  | Horses | 1963-2008 | Global | N2 |  |
| h3.2.1 | A/equine/SaoPaulo/6/1963(H3N8) | Horses | 1963-1972 | Global | N2 |  |
| h3.2.2 | A/equine/Kentucky/1/1987(H3N8) | Most: horses | 1976-2008 | Global | N2 | Some exceptions were from dogs in USA. |
| h3.3 |  | Horse and pigs |  |  |  | Few isolates were available. |
| h3.3.1 | A/equine/Argentina/1/2001(H3N8) | Horses | 2001 | Argentina | N8 | Few isolates were available. |
| h3.3.2 | A/swine/Quebec/4001/2005(H3N2) | Pigs | 2005 | Canada | N2 | Few isolates were available. |
| *h4.1* | *A/chicken/Alabama/1/1975(H4N8)* | *Major: birds*  *Minor: seals* | *1975-2004* | *Most: North America* | *Multiple* | *One exception was from Europe.* |
| *h4.2* | *A/duck/HongKong/24/1976(H4N2)* | *Birds* | *1956-2001* | *Eastern Hemisphere* | *Multiple* |  |
| h5.1 |  | Birds |  | Most: North America |  |  |
| h5.1.1 | A/turkey/Ontario/7732/1966(H5N9) | Birds | 1966-1975 | North America | N6, N9 |  |
| h5.1.2 | A/Chicken/Florida/2507/89(H5N2) | Birds | 1983-1989 | USA | N2 |  |
| h5.1.3 | A/duck/NewZealand/41/1984(H5N2) | Birds | 1984 | New Zealand | N2 |  |
| h5.1.4 | A/mallard/Wisconsin/428/75(H5N1) | Birds | 1975-2000s | Most: North America | Multiple | An exception was from Japan. |
| h5.1.5 | A/chicken/TX/1672804/02 | Birds | 2000s | USA |  |  |
| h5.1.6 | A/chicken/Mexico/232/94(H5N2) | Birds | 1994-2004 | Most: Mexico and Central America | N2 | Some exceptions were from Asia and USA. |
| h5.2 |  | Birds | 1959-2008 | Eastern Hemisphere | Multiple |  |
| h5.2.1 | A/chicken/Scotland/1959(H5N1) | Birds | 1959, 1961 | Eastern Hemisphere | N1, N3 |  |
| h5.2.2 | A/duck/JiangXi/6146/2003(H5N3) | Birds | 1976-2007 | Eastern Hemisphere | Multiple |  |
| h5.2.3 | A/goose/Guangdong/1/96(H5N1) | Major: birds | 1996-2008 | Eastern Hemisphere | N1 |  |
| h5.3 | A/Turkey/Ramon/73(H5N2) | Bird | 1973 | Roman | N2 | Only one virus has been identified within h5.3. |
| *h6.1* | *A/Turkey/Canada/63(H6N2)* | *Birds* | *1963-2002* | *Most: North America* | *Multiple* | *Two exceptions were from Australia.* |
| *h6.2* | *A/duck/Taiwan/0526/72(H6N1)* | *Birds* | *1972-2007* | *Major: Eastern Hemisphere* | *Multiple* | *A substantial part of isolates were from North America.* |
| h7.1 |  | Major: birds  Minor: humans | 1971-2007 | Western Hemisphere | Multiple |  |
| h7.1.1 | A/Turkey/Oregon/1971(H7N3) | Major: birds  Minor: humans | 1971-2007 | North America | Multiple |  |
| h7.1.2 | A/Turkey/Chile/4418/02(H7N3) | Birds | 2001-2002 | South America | N3 |  |
| h7.2 |  | Major: birds  Minor: humans | 1902-2007 | Eastern Hemisphere | Multiple |  |
| h7.2.1 | A/Chicken/Victoria/75(H7N7) | Birds | 1975-2007 | Australia | Multiple |  |
| h7.2.2 | A/Fowl/Rostock/1934(H7N1) | Birds | 1902-1945 | Eastern Hemisphere | Major:N1,N7 |  |
| h7.2.3 | A/Turkey/England/1963(H7N3) | Major: birds  Minor: humans | 1963-2007 | Eastern Hemisphere | Multiple |  |
| h7.3 | A/Equine/Prague/1/1956(H7N7) | Horses | 1956-1977 | Global | N7 | The equine viruses have not been found since the end of the 1970s. |
| h8 | A/Turkey/Ontario/6118/1968(H8N4) | Birds | 1968-2005 | Most: North America | Major:N4  Minor:N2 | One exception was from China, and few isolates were available. |
| h9.1 | A/Turkey/Wisconsin/1/1966 | Birds | 1960s | USA | N2 | An odd strain within h9.1 was isolated from China in 2000. |
| h9.2 | A/Quail/Arkansas/29209-1/93 | Birds | 1993-1996 | USA | N2 |  |
| h9.3 |  | Major: birds  Minor: pigs | 1976-2006 | Global | Major:N2 |  |
| h9.3.1 | A/Duck/HongKong/86/76(H9N2) | Birds | 1976-1984 |  | Major:N2 |  |
| h9.3.2 | A/goose/MN/5733-1/1980(H9N2) | Birds | 1980-1997 | North America | Major:N2 |  |
| h9.3.3 | A/Turkey/Germany/R90/95(H9N2) | Major: birds  Minor: pigs | 1993-2006 | Eastern Hemisphere | Major:N2 |  |
| h9.4 |  | Major: birds | 1988-2007 | Eastern Hemisphere | N2 | Some exceptions were from humans and pigs. |
| h9.4.1 | A/Quail/HongKong/G1/97 | Major: birds  Minor: humans | 1997-2007 | Eastern Hemisphere | N2 |  |
| h9.4.2 | A/chicken/Beijing/1/94 | Major: birds  Minor: humans | 1988-2007 | China and Japan | N2 |  |
| *h10.1* | *A/mallard/Ohio/99/1989(H10N7)* | *Birds* | *1977-2007* | *North America* | *Multiple* |  |
| *h10.2* | *A/chicken/Germany/n/1949(H10N7)* | *Most: birds* | *1949-2006* | *Eastern Hemisphere* | *Multiple* | *One exception was from a mink.* |
| h11.1 | A/Memphis/546/1974(H11N9) | Birds | 1974-2007 | Major: North America | Multiple | Few isolates were available. Some exceptions were from Asia. |
| h11.2 | A/duck/Yangzhou/906/2002(H11N2) | Birds | 1956-2004 | Eastern Hemisphere | Multiple | Few isolates were available. |
| *h12.1* | *A/duck/Alberta/60/1976(H12N5)* | *Birds* | *1976-2005* | *North America* | *Multiple* | *Few isolates were available.* |
| *h12.2* | *A/duck/Primorie/3691/02(H9N2)* | *Birds* | *1981-2002* | *Russia and Australia* | *Major:N9*  *Minor:N2* | *Few isolates were available.* |
| h13.1 | A/gull/Minnesota/945/1980(H13N6) | Major: birds  Minor: whales | 1980-2004 | North America | Multiple | Few isolates were available. |
| h13.2 | A/black-headed gull/Astrakhan/227/84(H13N6) | Birds | 1984-2000 | Europe | N6, N8 | Few isolates were available. |
| h13.3 | A/gull/Maryland/704/1977(H13N6) | Birds | 1977, 1998 | USA and Russia | N6 | Few isolates were available. |
| *h14* | *A/Mallard/Gurjev/263/82(H14N?)* | *Birds* | *1982* | *Russia* |  | *Few isolates were available.* |
| h15 | A/duck/Australia/341/83(H15N8) | Birds | 1979-1983 | Australia | Multiple | Few isolates were available. |
| *h16* | *A/shorebird/NewJersey/840/1986(H16N3)* | *Birds* | *1975-2006* | *North America and Europe* | *N3* | *Few isolates were available.* |
