# Supplementary material for: Panorama Phylogenetic Diversity and Distribution of Type A Influenza Virus
Source: PLoS One. 2009 Mar 27;4(3):e5022. doi: 10.1371/journal.pone.0005022 (PMC2658884; doi:10.1371/journal.pone.0005022)
Supplement: Table S2 — Distribution of the lineages and sublineages within subtypes N1–N9 influenza viruses (0.15 MB DOC) [file pone.0005022.s002.doc]

**Supporting Information 3:**

**The hosts, isolation periods, isolation places and HA subtypes of the lineages and sublineages within subtypes N1~N9.**

| **Lineage/ sublineage** | **Some representatives** | **Host** | **Isolation period** | **Isolation place** | **HA subtype** | **Other information** |
| --- | --- | --- | --- | --- | --- | --- |
| n1.1 |  | Major: birds | 1934-2008 | Global | Multiple | A few isolates were from mammals. |
| n1.1.1 | A/FPV/Rostock/34(H7N1) | Birds | 1934 | Germany | H7 |  |
| n1.1.2 | A/gull/Delaware/2952/88(H11N1) | Birds | 1985-1993 | North America | Multiple |  |
| n1.1.3 | A/duck/AUS/749/1980(H1N1) | Birds | 1979-1984 | Oceania | Multiple |  |
| n1.1.4 | A/duck/NJ/771770/1995(H1N1) | Birds | 1976-2007 | Most: Western Hemisphere | Multiple | One exception was from Hong Kong. |
| n1.1.5 | A/chicken/Scotland/1959(H5N1) | Birds | 1959-1987 | Most: Eastern Hemisphere | Major: H6  Minor: H5 | One exception was from North America. |
| n1.1.6 | A/mallard/Stralsund/41-6/81(H2N1) | Birds | 1973-1981 | Eastern Hemisphere | Multiple |  |
| n1.1.7 | A/swine/Belgium/1/83(H1N1) | Most: Pigs | 1982-2007 | Eastern Hemisphere | H1 | Two exceptions were avian and human viruses, respectively. |
| n1.1.8 | A/duck/HongKong/716/1979(H6N1) | Birds | Most: 1979-1980 | Eastern Hemisphere | Multiple | One exception was isolated in 2001. |
| n1.1.9 | A/HongKong/156/97(H5N1) | Major: birds | 1997-2005 | Eastern Hemisphere | Multiple | The avian viruses caused 18 human severe infections Hong Kong in 1997. |
| n1.1.10 | A/Mallard/France/D710/02(H1N1) | Birds | 1996-2006 | Eastern Hemisphere | Multiple |  |
| n1.1.11 | A/Goose/Guangdong/1/96(H5N1) | Major: birds  Minor: mammals | 1996-2008 | Eastern Hemisphere | H5 | The H5N1 viruses of this sublineage were the HPAI ones circulating in the Eastern Hemisphere in recent years. |
| n1.2 |  | Major: humans | 1918-2008 | Global | H1 |  |
| n1.2.1 | A/BrevigMission/1/1918(H1N1) | Humans | 1918 | Global | H1 |  |
| n1.2.2 | A/WSN/1933(H1N1) | Most: humans | Most: 1933-1947 | Global | H1 | A few exceptions were swine. |
| n1.2.3 | A/Fiji/15899/83(H1N1) | Most: humans | Most: 1948-2000 | Global | H1 | Two exceptions were swine isolated after 2003. |
| n1.2.4 | A/Thailand/39/2008(H1N1) | Most: humans | 1995-2008 | Global | H1 | Some exceptions were from birds, pigs or giant anteaters. |
| n1.3 |  | Major: pigs | 1930-2007 | Global | Major: H1  Minor: H3 |  |
| n1.3.1 | A/swine/Iowa/15/1930(H1N1) | Pigs | Most: 1930-1945 | Global | H1 | One exception was isolated in 1973. |
| n1.3.2 | A/swine/Kyoto/3/1979(H1N1) | Most: pigs  Some: humans and birds | 1957-2007 | Global | Major: H1  Minor: H3 | Previously, this lineage was designated as “classical swine H1 lineage”. |
| *n2.1* |  | *Most: birds* |  |  |  |  |
| *n2.1.1* | *A/Turkey/Canada/63(H6N2)* | *Birds* | *Most: 1963-1986* | *Most: Eastern Hemisphere* | *Multiple* | *One exception was from China in 2002 and another exception was from Canada in 1963* |
| *n2.1.2* | *A/Turkey/England/69* | *Bird* | *1969* | *England* | *H3* |  |
| *n2.1.3* | *A/Turkey/Wisconsin/1/1966(H9N2)* | *Birds* | *1966, 1975* | *USA* | *H5, H9* |  |
| *n2.1.4* | *A/chicken/Beijing/1/94* | *Most: birds* | *1994-2007* | *Eastern Hemisphere* | *H9* | *A few viruses within this sublineage were from pigs and humans.* |
| *n2.1.5* | *A/Quail/HongKong/AF157/92* | *Bird* | *1992* | *Hong Kong* | *H9* |  |
| *n2.1.6* | *A/duck/Nanchang/1749/1992(H11N2)* | *Most: birds*  *One: pigs* | *Most:1992-2007* | *Eastern Hemisphere* | *Multiple* | *One exception was isolated in 1979.* |
| *n2.1.7* | *A/turkey/Massachusetts/3740/1965(H6N2)* | *Major: birds*  *Minor: human* | *1965-2006* | *Major: Western Hemisphere* | *Multiple* | *One clade within this sublineage was from Eastern Hemisphere.* |
| *n2.2* |  | *Most: human or pigs* | *1957-2008* | *Global* |  | *Only a few isolates were avian* |
| *n2.2.1* | *A/Guiyang/1/1957(H2N2)* | *Major: human*  *Minor: pigs* | *Most: 1957-1989* | *Global* | *Major: H2*  *Minor H3* | *An exception was isolated in 2005.* |
| *n2.2.2* | *A/swine/Miyagi/5/03(H1N2)* | *pigs* | *1980-2006* | *Eastern Hemisphere* | *H1* |  |
| *n2.2.3* | *A/sw/Gent/1/84(H3N2)* | *Most: pigs* | *1984-2006* | *Eastern Hemisphere* | *H1, H3* | *Several exceptions were isolated from humans.* |
| *n2.2.4* | *A/Victoria/1/1968(H3N2)* | *Major: humans and pigs* | *1968-2008* | *Global* | *Major: H3*  *Some: H1* | *Several exceptions were avian.* |
| n3.1 |  | Birds | 1961-2007 | Global | Multiple |  |
| n3.1.1 | A/turkey/CO/13356/91(H7N3) | Most: Birds | 1971-2007 | Western Hemisphere | Multiple | Some viruses within this sublineage were isolated from mammals. |
| n3.1.2 | A/turkey/England/1963(H7N3) | Birds | 1961-2007 | Eastern Hemisphere | Multiple |  |
| n3.1.3 | A/chicken/Chile/4322/02(H7N3) | Birds | 2002 | Chile | H7 | Only one representative was available |
| n3.2 | A/tern/Astrakan/775/83(H13N3) | Birds | 1975-2006 | Global | Multiple |  |
| *n4.1* | *A/mallardduck/ALB/7/1987(H8N4)* | *Birds* | *1968-2007* | *North America* | *Multiple* |  |
| *n4.2* | *A/duck/Hokkaido/18/00(H10N4)* | *Most: Birds* | *1979-2006* | *Eastern Hemisphere* | *Multiple* | *An exception was isolated from a mink.* |
| n5.1 | A/mallard/MN/280/1999(H3N5) | Birds | 1976-2005 | North America | Multiple |  |
| n5.2 | A/duck/Mongolia/149/03(H10N5) | Birds | 1972-2004 | Eastern Hemisphere | Multiple |  |
| *n6.1* | *A/mallard/Ohio/298/1987(H4N6)* | *Most: birds* | *1976-2006* | *Most: North America* | *Multiple* | *One exception was from a pig and another was from Asia.* |
| *n6.2* | *A/duck/England/1/1956(H11N6)* | *Birds* | *1956-2007* | *Major: Eastern Hemisphere* | *Multiple* | *Some viruses were isolated from North America.* |
| n7.1 | A/mallard/Ohio/99/1989(H10N7) | Most: birds | 1977-2007 | North America | Multiple | An exception was isolated from a seal. |
| n7.2 |  | Most: birds | 1934-2006 | Eastern Hemisphere | Major: H7 |  |
| n7.2.1 | A/fowl/Dobson/1927(H7N7) | Birds | 1927-1934 | England and USA | H7 | Few viruses were available. |
| n7.2.2 | A/chicken/Victoria/1976(H7N7) | Birds | 1949-1993 | Eastern Hemisphere | Major: H7 |  |
| n7.2.3 | A/chicken/Netherlands/1/03(H7N7) | Most: birds | 1997-2006 | Eastern Hemisphere | Multiple | Some exceptions were isolated from humans. |
| n7.3 | A/equine/Prague/1/1956(H7N7) | Most: equine | 1956-1992 | Global | Most: H7 | One exception was swine. |
| *n8.1* | *A/shorebird/DE/12/2004(H6N8)* | *Birds* | *1963-2007* | *Major: North America* | *Multiple* | *Some viruses were from Eastern Hemisphere.* |
| *n8.2* | *A/turkey/Ireland/1378/1983(H5N8)* | *Most: birds* | *1963-2006* | *Eastern Hemisphere* | *Multiple* | *An exception was isolated from a horse.* |
| *n8.3* | *A/equine/Kentucky/2/1980(H3N8)* | *Most: horses* | *1963-2005* | *Global* | *H3* | *Some exceptions were isolated from dogs.* |
| n9.1 |  | Most: birds | 1974-2007 | Major: North America | Multiple | An exception was isolated from a whale. |
| n9.1.1 | A/gull/MD/19/1977(H2N9) | Birds | 1974-2007 | Most: North America | Multiple |  |
| n9.1.2 | A/shorebird/Korea/S8/2006(H11N9) | Birds | 1996-2006 | Eastern Hemisphere | Multiple |  |
| n9.2 | A/chicken/Italy/22A/1998(H5N9) | Birds | 1978-1999 | Most: Eastern Hemisphere | Multiple | Some exceptions were from North America. |
