# Supplementary material for: Panorama Phylogenetic Diversity and Distribution of Type A Influenza Virus
Source: PLoS One. 2009 Mar 27;4(3):e5022. doi: 10.1371/journal.pone.0005022 (PMC2658884; doi:10.1371/journal.pone.0005022)

**Text S1. The impact of intermediate strains on phylogenetic classification.**

The impact was demonstrated by the following figure.

Distinct three clades (Part A in the figure) became difficult to be classified due to the existence of some intermediate strains (Part B in the figure). Bootstrap values at the nodes where intermediate strains located are usually low because it is of some possibility for the intermediate strains to be located at other branches. Both of the two classifications (Part C and D in the figure) for the isolates were possibly rational, although also with some subjectivity and uncertainty.


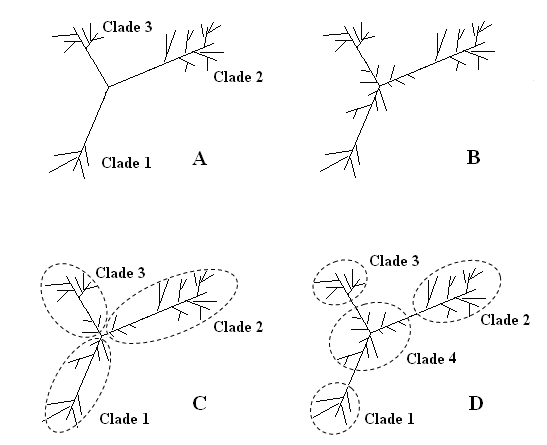

Supplement: Text S1 — The impact of intermediate strains on phylogenetic classification (0.04 MB DOC) [file pone.0005022.s003.doc]
