# Supplementary material for: Panorama Phylogenetic Diversity and Distribution of Type A Influenza Virus
Source: PLoS One. 2009 Mar 27;4(3):e5022. doi: 10.1371/journal.pone.0005022 (PMC2658884; doi:10.1371/journal.pone.0005022)

**Text S2. Panorama phylogenetic distribution of the viruses in sublineage h5.2.3 in 2005~2008 based on 132 representatives.**

The distribution was showed by the figure in the next page.

The 132 representatives of the viruses within the sublineage isolated in 2005~2008 were selected by randomly choosing one from those which were similar to each other (approximately genetic distances <1.6%), isolated in the same year and from the same province of China or the same country other than China (as a China province is nearly as large as a country other than China in the Eastern Hemisphere). Bootstrap values were given at relevant nodes in the figure.

The clades and subclades of the viruses were classified according to the WHO/FAO/OIE nomenclature. The viruses marked with triangles have not been classified by the WHO/FAO/OIE nomenclature. The figure validated the WHO/FAO/OIE nomenclature, and found that four subclades, 2.1.4, 2.1.5, 2.16 and 2.3.5, in the figure were missed or unclassified by the WHO/FAO/OIE nomenclature.

Some clades (3, 5, 6, 8) within the WHO/FAO/OIE nomenclature were not shown in the figure as they have not been reported after 2004, and possibly have been replaced by new clades. Furthermore, six clusters (1, 4+7, 9, 2.1, 2.2 and 2.3) were identified circulating in more than one place and more than one year. Each of the six clusters corresponded to a certain region in the Eastern Hemisphere, which indicated that the viruses possibly have become endemic in these regions.

The figure also demonstrated that the diversity of the H5N1 subtype HPAI viruses circulating in China and Indonesia was significantly higher than elsewhere, which indicated the HPAI viruses possibly have become more entrenched in these two countries.


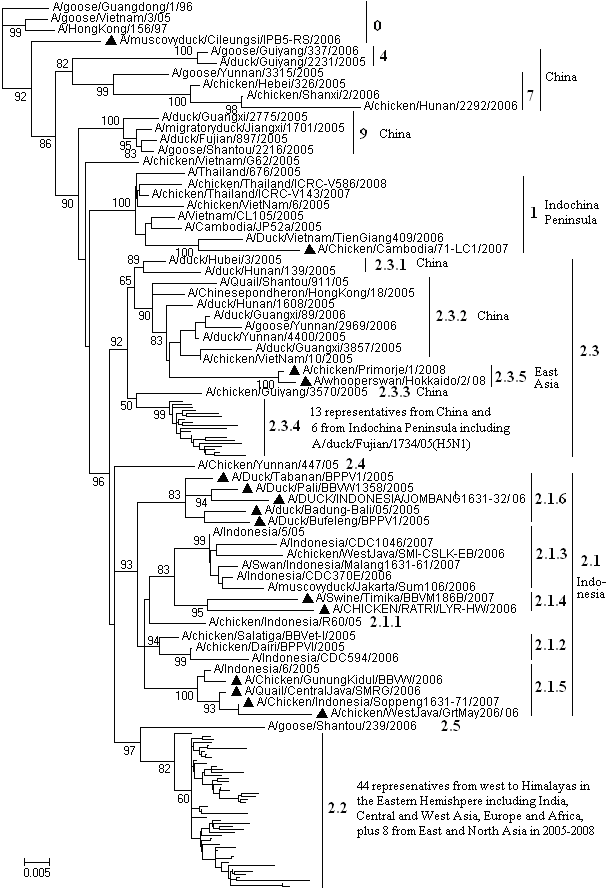

Supplement: Text S2 — Panorama phylogenetic distribution of the viruses in sublineage h5.2.3 in 2005∼2008. (0.08 MB DOC) [file pone.0005022.s004.doc]
